# Gastric perforation with foreign body granuloma formation caused by a short hair—a case report

**DOI:** 10.3389/fped.2025.1521428

**Published:** 2025-03-17

**Authors:** Gang Shen, Yunpeng Zhai, Huashan Zhao, Rui Guo, Hongxiu Xu, Sai Huang, Shisong Zhang

**Affiliations:** Department of Thoracic and Oncology Surgery, Children's Hospital Affiliated to Shandong University, Jinan Children's Hospital, Jinan, China

**Keywords:** hair, gastric perforation, foreign body granuloma, surgical treatment, child

## Abstract

**Objective:**

This case report presents the diagnosis and treatment process of a rare case of gastric perforation caused by a short hair, leading to the formation of a foreign body granuloma in a child.

**Case Report:**

The patient was a 3-year-old boy who was admitted to the hospital with persistent abdominal pain and fever for more than 20 days. Ultrasound and CT revealed a foreign body and inflammatory encapsulation in the abdominal cavity. Laparoscopic and open surgeries were performed, revealing a full-thickness gastric wall perforation approximately 2 mm in diameter on the greater curvature side of the stomach, which was in contact with the abscess and contained purulent fluid and short hair approximately 1.5 cm long. The intraoperative diagnosis was hair-induced gastric perforation, leading to intra-abdominal foreign body inflammatory granuloma. Granuloma excision, gastric perforation repair, and partial transverse colon resection were performed. Postoperative pathological results revealed a gastric perforation with surrounding acute and chronic inflammation, and the diagnosis was a reactive fibrous granulomatous lesion. The patient recovered well after surgery, and follow-up for one year revealed no significant abnormalities.

**Conclusion:**

Hair-induced gastric perforation leading to a foreign body granuloma formation is a rare disease. Imaging examinations play a key role in diagnosis, and surgical resection is the main treatment method.

## 1 Introduction

Abdominal foreign body granulomas are chronic inflammatory reactions caused by foreign bodies that enter the body from the outside, usually manifesting as the formation of granulation tissue in local areas. These granulomas can be caused by various types of foreign bodies, including surgical sutures, surgical sponges, fish bones, gauze, gallstones, etc (1). Foreign body granulomas can occur in the gastrointestinal tract, abdominal cavity, liver, and other sites and may be difficult to differentiate from other types of tumors or lesions, such as gastric cancer and liver metastasis (2).

Foreign body granulomas in the abdominal cavity are often caused by gastrointestinal perforation. Due to the fine and soft characteristics of hair, cases caused by hair perforation are extremely rare. They usually do not cause significant gastrointestinal symptoms, and due to their difficulty in being detected by related examinations, the diagnosis is challenging. However, once hair penetrates the gastric wall, it may trigger a local inflammatory response, leading to the formation of a granuloma, and may cause a series of complications, including infection symptoms, bleeding, and obstruction.

This case report presents the diagnosis and treatment process of a patient with gastric perforation caused by short hair that eventually led to the formation of a foreign body granuloma. With this case, we hope to increase clinical awareness of such rare conditions and provide references for future diagnosis and treatment.

## 2 Case report

### 2.1 General situation

**Figure 1:**
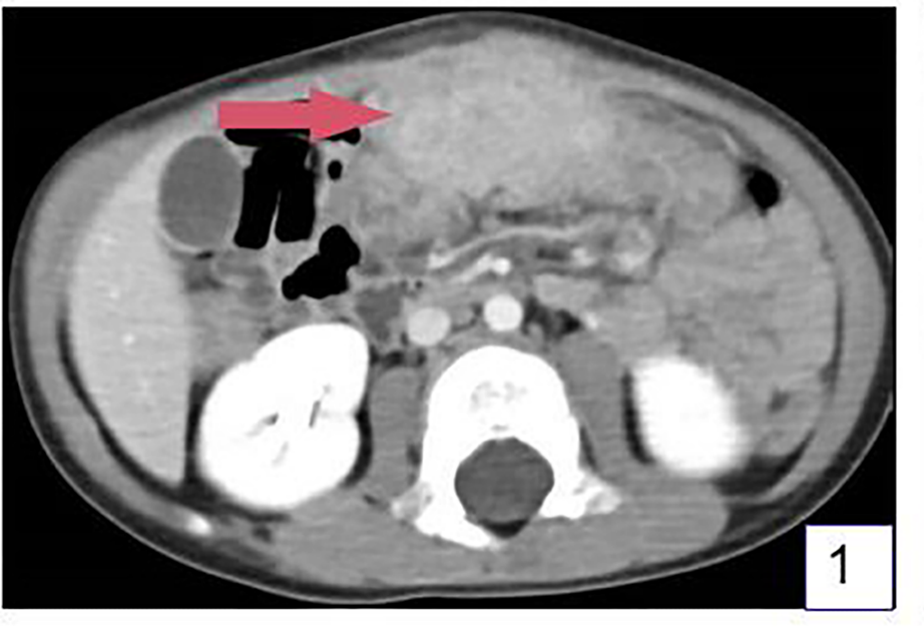
Ct examination revealed a lump under the anterior abdominal wall of the upper abdomen, about 6 cm*4cm*3 cm in size, with uneven density and unclear boundary. (Arrow shows the mass).

**Figure 2:**
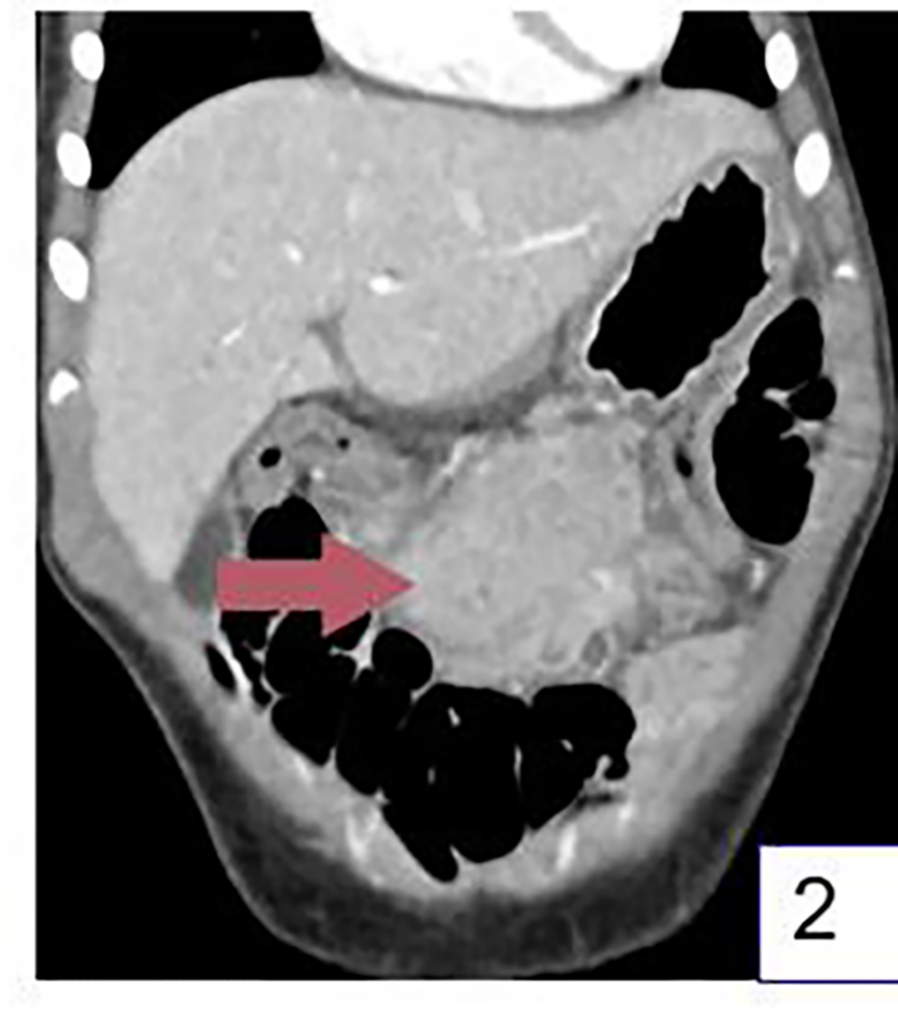
Coronal CT images showed the mass located between the stomach and colon. (Arrow shows the mass).

**Figure 3:**
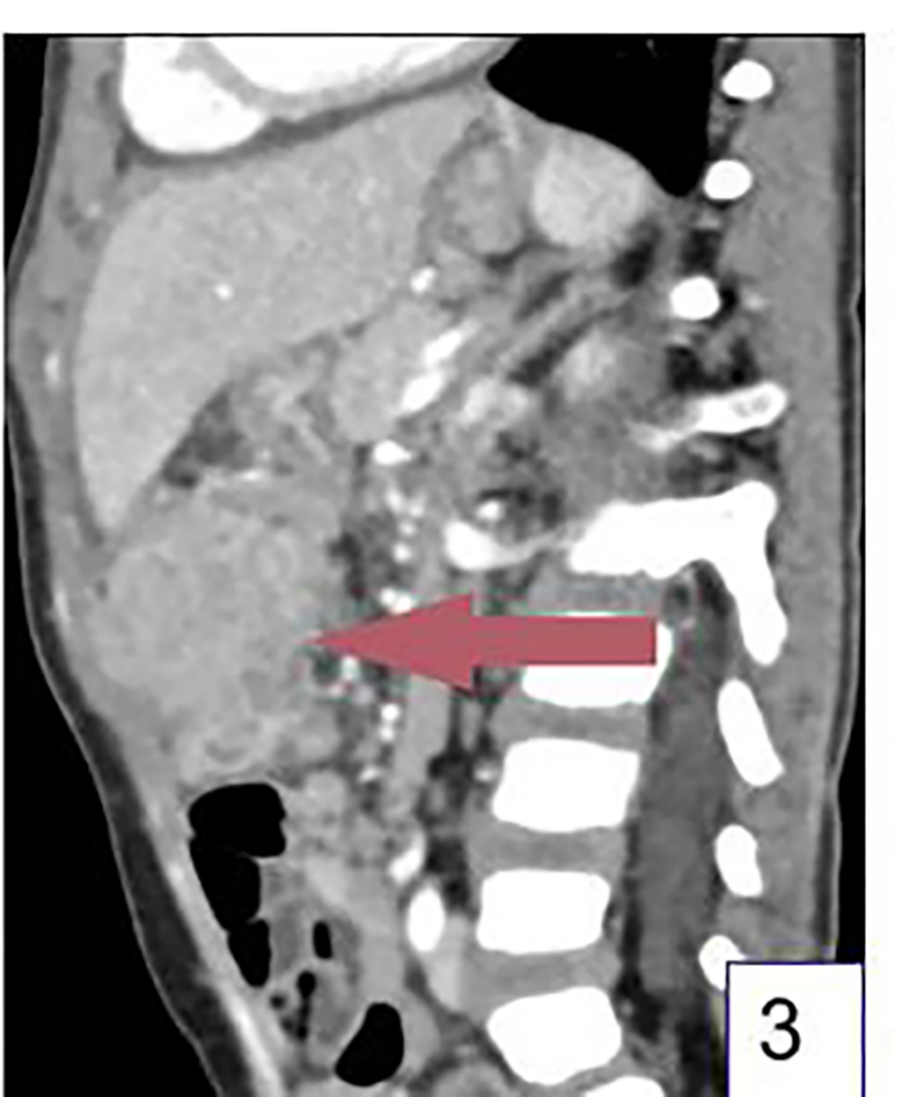
Sagittal CT image. (Arrow shows the mass).

The patient was a 3-year-old boy who was admitted to the hospital due to abdominal pain for more than 20 days. More than 20 days before admission, the patient developed abdominal pain after eating a large amount of food. The pain was intermittent, located in the upper abdomen, and worsened at night, accompanied by fever with a maximum body temperature of 38.1°C. The fever subsided after the patient took cefixime granules orally self-administered, but the patient's abdominal pain was not significantly alleviated. The patient took cefixime granules intermittently during this period. Five days before admission, the patient's abdominal pain worsened, with frequent episodes of pain, and fever recurred. An upper abdominal CT scan at a local hospital revealed an abdominal tumor, inflammatory myofibroblastoma was initially considered for diagnosis (Figures 1–3). And abdominal ultrasound examination suggested the possibility of a foreign body in the abdominal cavity with surrounding inflammatory encapsulation. The patient was then referred to our hospital. The patient had no special medical history, and personal and family histories were unremarkable.

Upon admission examination, the abdomen was flat without distension, no gastrointestinal type or peristaltic waves were observed, mild tenderness in the upper abdomen was observed, there was no rebound tenderness, and a mass about 6 cm*4 cm*3 cm in size was palpable in the upper abdomen, with a tough texture and tenderness. The liver and spleen were not palpable below the costal margin, there was no shifting dullness, and there were 4 bowel sounds/minute.

### 2.2 Auxiliary examination

**Figure 4:**
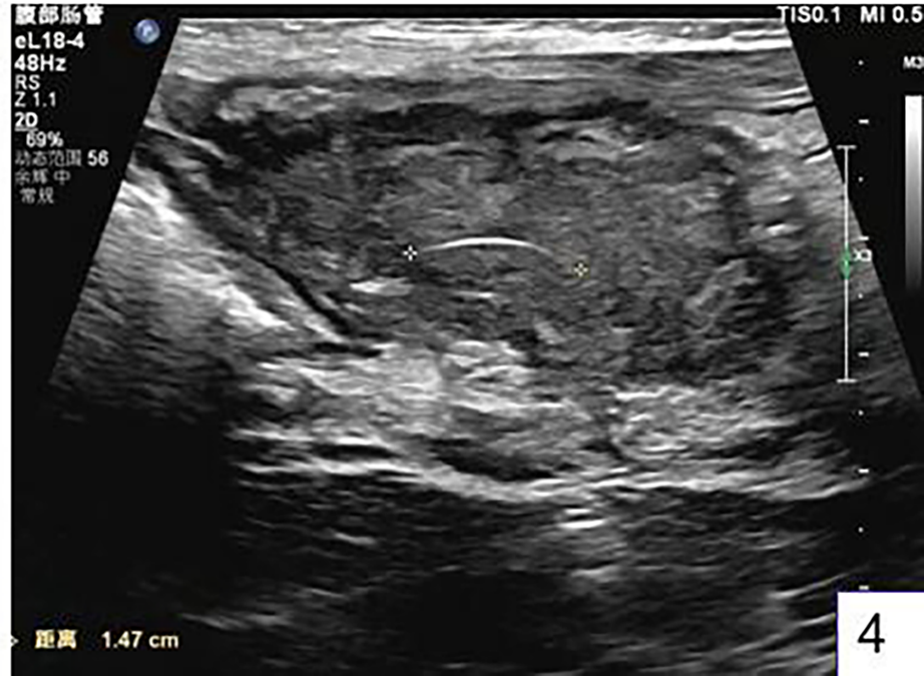
Color ultrasound examination found that there was pus in the mass, and a curved foreign body about 1.6 cm in length was found in the pus cavity.

After admission, the patient underwent a follow-up ultrasound, which revealed thickening of the greater omentum in the upper abdomen, a reduced echo, measuring approximately 5.7 cm × 3.8 cm × 2.8 cm in size, with irregular edges, and a length of approximately 1.6 cm arc-shaped strong echo was visible inside, with rich blood flow signals on color Doppler imaging (CDFI). The intestinal peristalsis in the abdominal cavity was normal, with no signs of dilation or fluid accumulation. The mesentery was normal. No obvious anechoic area was detected in the abdominal or pelvic cavities (Figure 4). The routine blood test results were as follows: white blood cell count, 18.68*10^9^/L*; red blood* cell count, 3.59*10^9^/L; hemoglobin, 91 g/L; platelet count, 438*10^9^/L*; neutrophil count, 15.*43*10^9^/L; neutrophil ratio, 82.60%; CRP, 10.79 mg/L; and ion biochemistry and liver and kidney function tests were all normal.

### 2.3 Intraoperative findings

**Figure 5:**
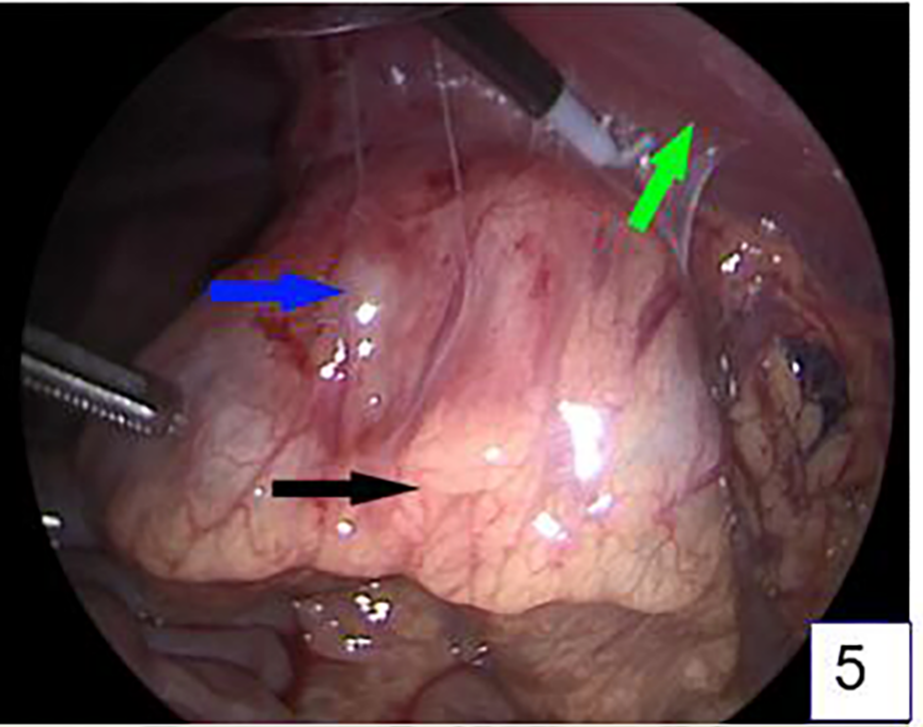
Laparoscopic exploration revealed a mass between the gastrocolon in the upper abdomen, about 6 cm*4cm*3 cm in size, which was closely adhered to the stomach, transverse colon, and anterior abdominal wall. (The blue arrow is the mass, the black arrow is the transverse colon, and the green arrow is the anterior abdominal wall).

**Figure 6:**
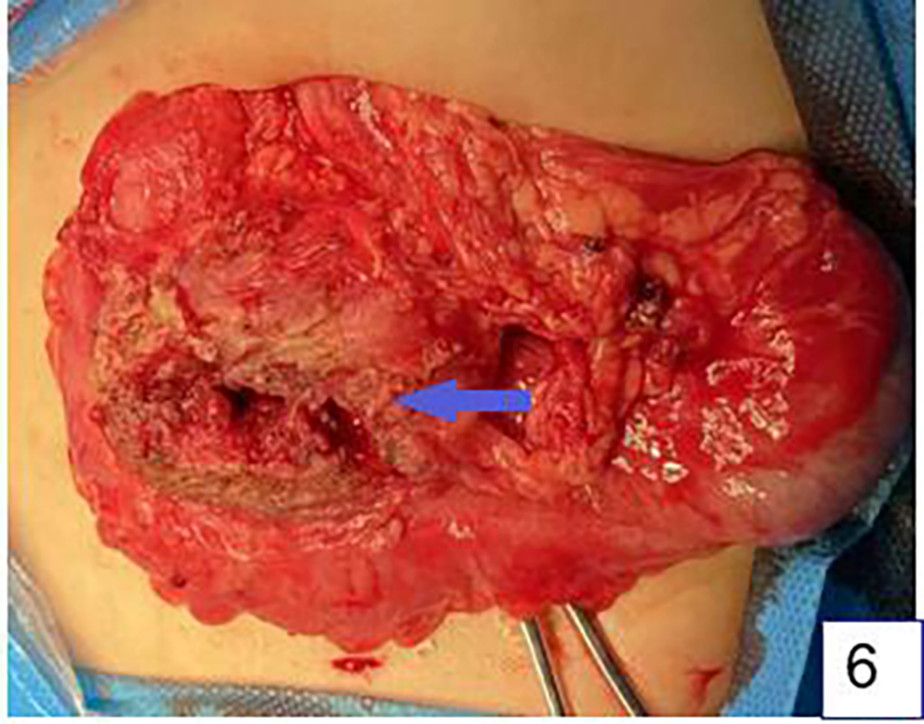
The situation of open surgery. There is a cystic mass (arrow) with a thick wall, a small amount of purulent fluid, a short hair, and a small perforation in the stomach wall. The mass is closely adherent to the colon.

**Figure 7:**
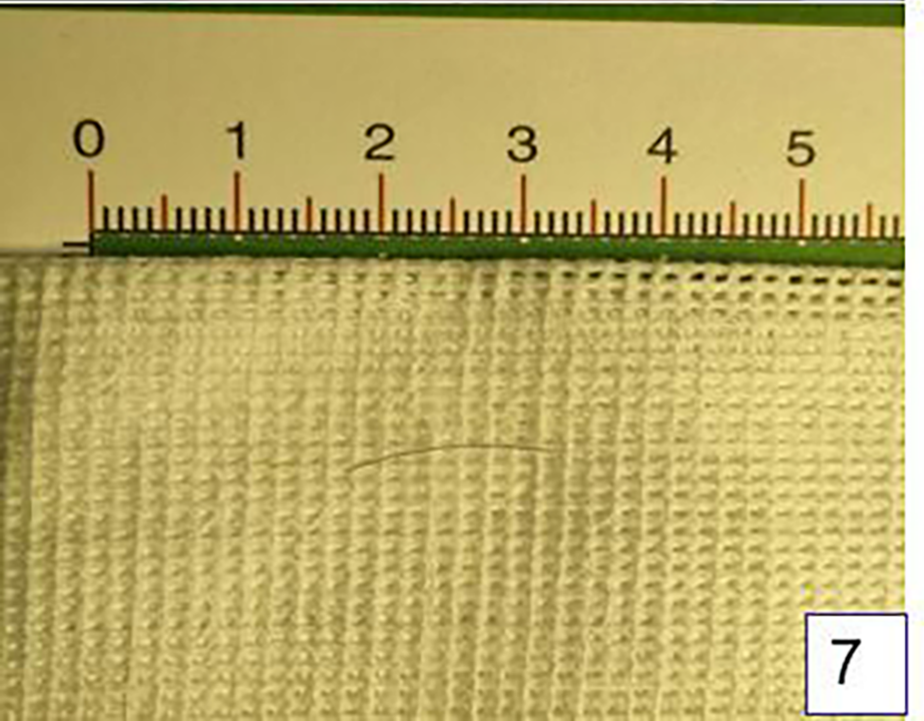
A short hair about 1.6 cm long was found during the operation.

The patient underwent surgery the day after admission. Laparoscopy was first performed to explore the abdominal cavity (Figure 5), with three 5 mm trocars placed in the umbilical, left lower abdomen, and right lower abdomen. Laparoscopic exploration revealed a mass approximately 6 cm*4 cm*3 cm in size between the greater curvature of the stomach and the transverse colon. The mass was severely adhered to the gastric wall, transverse colon, and abdominal wall, with part of the greater omentum wrapping around the mass. Attempts to separate the mass laparoscopically were difficult, so an open laparotomy was performed. An approximately 7 cm long incision was made in the right upper abdomen, and the mass was separated from the abdominal wall. The mass, along with the adherent gastric wall and colon, was removed from the abdominal cavity (Figure 6). The mass was separated first from the gastric wall, and a full-thickness perforation approximately 2 mm in diameter on the greater curvature side of the stomach was observed, which was in contact with the inside of the mass. Purulent fluid was found inside the mass, and a hair approximately 1.6 cm long was identified (Figure 7). The intraoperative diagnosis was hair-induced gastric perforation leading to a foreign body inflammatory granuloma. A small amount of gastric tissue around the perforation was trimmed, and the gastric perforation was repaired with full-layer interrupted sutures. The mass was separated from the transverse colon, and the inflammation had spread to the serosa and mucosal layer of the transverse colon, making the adhesion inseparable. With the consent of the family, the granuloma and part of the transverse colon were resected, and the two ends of the colon were anastomosed with full-layer interrupted sutures. The repair site of the gastric perforation and the intestinal anastomosis site were checked for no air leakage, the abdominal cavity was irrigated with warm saline, and a drainage tube was left before the abdomen was closed to end the surgery.

### 2.4 Postoperative course

**Figure 8:**
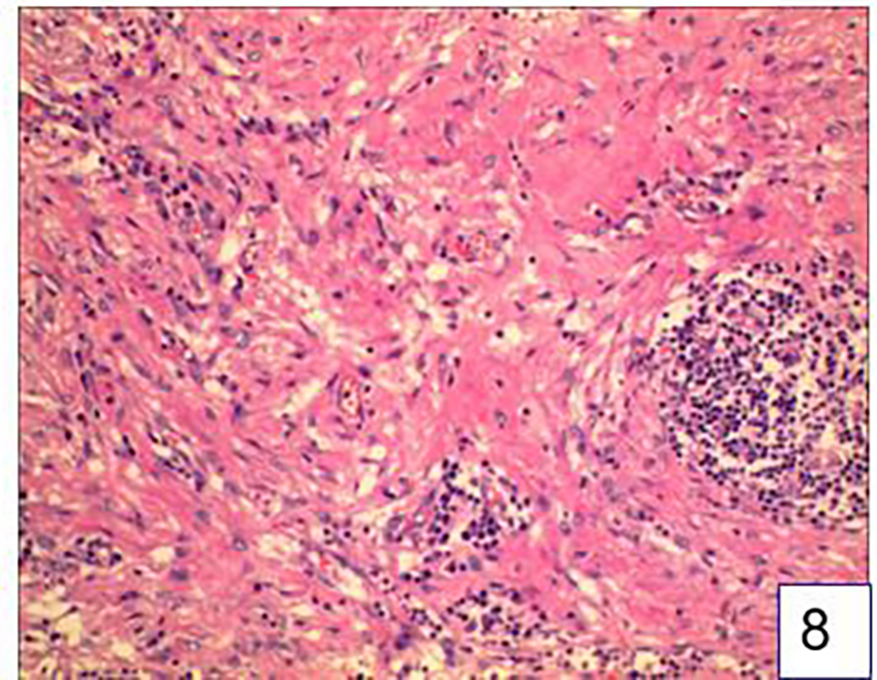
Postoperative pathology confirmed that the mass was foreign body granuloma.

Microscopic findings: Macrophages, lymphocytes, and plasma cells were aggregated in the lesion area, accompanied by infiltration of neutrophils and eosinophils. The lesion site showed significant granulation tissue proliferation, abundant new capillaries, epithelioid cell aggregation, and a large number of fibroblasts and fibroblasts with disordered arrangement. The final pathological diagnosis was gastric perforation with peripheral acute and chronic inflammation and reactive fibrous granulomatous lesions. (Figure 8). The patient was given cefazolin for anti-inflammatory treatment and parenteral nutrition support and gradually returned to a regular diet. The patient was discharged on the 7th day after surgery. Follow-up at 1 month, 3 months, and 1 year postoperatively revealed that the patient had recovered well, with no significant abnormalities observed.

## 3 Discussion

Gastric perforation refers to the destruction of the integrity of the gastric wall, leading to leakage of the gastric contents into the abdominal cavity. This condition can be caused by various factors, including peptic ulcers, tumor erosion, trauma, or foreign body penetration (3). Foreign bodies that cause gastric perforation are generally sharp and hard objects, including bamboo, metal nails, glass fragments, and fish bones (4). As reported in this case, hair as a foreign body causing gastric wall perforation is a rare cause, and we have not found any previous reports in our database.

Analyzing how a soft, short hair can penetrate the gastric wall may be related to the local pathological state of the patient's gastric wall at the time. Before the patient developed gastric perforation, they consumed a large meal, which could lead to gastric dilation and subsequent thinning of the gastric wall, making it easier for the hair to penetrate the gastric wall. Other possible reasons include the presence of inflammation or ulcers in the gastric wall at the time, which could weaken the gastric wall, making it easier for sharp foreign bodies to penetrate, and even blunt foreign bodies could penetrate the gastric wall (5).

In this case, after the hair penetrated the gastric wall, due to its indigestibility and local inflammatory response, a foreign body granuloma formed in the abdominal cavity. This mainly involves the immune response and tissue repair process caused by foreign bodies in the abdominal cavity (6). When a foreign body enters the abdominal cavity, the immune system triggers an inflammatory response by releasing cytokines to recruit macrophages and lymphocytes, aiming to eliminate the foreign material. These immune cells secrete enzymes and growth factors that stimulate fibroblast proliferation, forming granulation tissue to encapsulate the foreign body and prevent further inflammation. If the foreign body persists long-term, the granulation tissue may develop into chronic granulomas, leading to localized tissue fibrosis, sclerosis, and potential complications^(^6).This process may be accompanied by local infection and infiltration of inflammatory cells, eventually leading to the formation of an encapsulated mass, which may appear as a mass with a specific density on imaging.

Immunologically, granuloma formation is an orderly process involving the activation, transformation, and recruitment of macrophages, which can undergo epithelioid transformation to form tightly connected cell membranes, similar to epithelial cells. Mature granulomas may undergo structural changes, such as fibrosis and necrosis, which may lead to tissue damage and disease (7). Foreign bodies cause immune response in local tissues due to physical or chemical stimulation. Under the microscope, a large number of macrophages, fibroblasts and foreign body giant cells can be seen in the lesion area around the core of the foreign body, forming nodular lesions with clear horizons, which may also be accompanied by infiltration of lymphocytes, neutrophils and eosinophils. This hyperplastic response is a defense mechanism used by the body to try to enclose or remove foreign bodies^(^8). The appearance of gastric foreign body granulomas may not be as clear as other types of granulomas, with relatively few inflammatory cells, while foreign bodies are more compact and often accompanied by significant fibrosis and calcification (9). In this case, we found a large number of immune cells, fibrocytes and fibroblasts under the microscope, which is consistent with the pathological manifestations of gastrointestinal foreign body granuloma.

In clinical practice, it is challenging to make a preoperative diagnosis of inflammatory granulomas caused by foreign bodies. First, the clinical manifestations of gastric perforation may be atypical, especially when it is caused by uncommon factors, such as hair. Patients may only exhibit nonspecific abdominal pain or discomfort, making early diagnosis complicated. Second, the formation of foreign body granulomas may further conceal the signs of the original perforation, leading to delayed diagnosis.

In such cases, imaging examinations become key to diagnosis. Ultrasound has limitations in diagnosing gastrointestinal perforation; it is not very useful for detecting free air without direct or indirect findings of pneumoperitoneum, but it can help confirm intestinal paralysis and free fluid in the abdominal cavity. However, foreign body granulomas in the abdominal cavity can be confirmed by examining the foreign body inside the granuloma (10). CT can help locate the perforation more precisely in the upper abdomen, middle abdomen, or lower abdomen by confirming the volume of free air and free fluid, and it can provide more accurate confirmation of the location and density of foreign bodies in the abdominal cavity (11).

However, even with these advanced diagnostic tools, the diagnosis of foreign body granulomas remains challenging. As noted by Chung et al. in their 2023 case report, foreign body perforation may lead to the formation of retroperitoneal abscesses, which can be clinically difficult to distinguish from tumors or other inflammatory diseases (12). Moreover, the presence of foreign bodies may be overlooked in imaging examinations, especially when the foreign bodies are small or hidden, such as the small hair foreign body in the granuloma center of the patient in this case, which was overlooked in the ultrasound examination and was difficult to distinguish in the CT images.

The treatment of abdominal foreign body granulomas requires comprehensive consideration of the characteristics, location, and complications caused by the foreign body. The main goal of treatment is to safely remove the foreign body, alleviate symptoms, and prevent further complications. Endoscopic treatment and surgery are the two main treatment methods, and antibiotic treatment is an important part of adjunctive treatment. During the treatment process, the patient's condition should be closely monitored, and the treatment plan should be adjusted according to the specific situation.

Endoscopic treatment is the preferred method for removing foreign bodies in the stomach, especially those that are small and have a regular shape (13). However, for foreign bodies that have caused perforation or granuloma formation, endoscopic treatment may not be applicable, and laparoscopic surgical exploration is needed. Laparoscopic surgery is an effective method for safely removing foreign bodies in the abdominal cavity and treating surrounding abscesses (1, 14). When foreign bodies cause perforation or complications, such as intestinal perforation and abscess formation, endoscopic therapy has certain limitations. For example, case reports indicate that endoscopic therapy is risky when perforation is suspected, and exploratory laparotomy is recommended to ensure safety. In addition, endoscopic removal of foreign bodies may not completely remove the foreign body stump and may damage neighboring organs^(^15). In this case, considering the uncertainty of preoperative diagnosis and the limitations of endoscopic treatment for granuloma, we chose direct surgery instead of endoscopy. Of course, in the past, there have been successful cases of using digestive endoscopy to treat the perforation of foreign bodies in the stomach. Whether foreign body granuloma can resolve after the removal of foreign bodies needs further observation.

For foreign bodies that have formed granulomas, surgical excision is necessary. The foreign body and granuloma can be completely removed by surgery, immediately alleviating symptoms, reducing the risk of infection spreading, and allowing histopathological examination for diagnosis. For granulomas with extensive adhesions or poor locations, surgery may be more complex. Laparoscopic technology can help manage inflammatory granulomas formed by gastrointestinal perforation, but complex granulomas still require open surgery for more thorough treatment (14, 16). For abscesses caused by foreign bodies, in addition to removing the foreign body, adequate drainage is needed. As described by Minh et al. in their 2023 case, laparoscopic surgery removed the fish bone, causing liver abscesses, and no signs of gastrointestinal perforation were found, indicating that foreign bodies may cause serious complications without obvious signs of perforation, requiring surgical intervention (17). Antibiotic treatment is necessary after foreign body removal to prevent or treat possible infections. As mentioned by Venkatesan et al. in their 2019 case, the patient received 8 weeks of antibiotic treatment after the removal of fish bones and abscess drainage (18). In this case, we removed a very small portion of the colon, because during the operation it was found that the granuloma and the transverse colon were extremely adhesive and could not be separated, and the transverse colon was eroded and thin. Granulomatous and segmental-transverse colectomy is performed only with the consent of the family, which can be avoided if the adhesions are successfully isolated.

In summary, this case demonstrates the entire process of diagnosis and treatment of an extremely rare case of gastric perforation caused by a short hair leading to the formation of an abdominal foreign body granuloma, showing that even soft short hair can cause gastric perforation and ultimately require surgical treatment.

## Data Availability

The original contributions presented in the study are included in the article/Supplementary Material, further inquiries can be directed to the corresponding author.
