# Supplementary figures and images for: Gastric perforation with foreign body granuloma formation caused by a short hair—a case report
Source: Front Pediatr. 2025 Mar 17;13:1521428. doi: 10.3389/fped.2025.1521428 (PMC11956502; doi:10.3389/fped.2025.1521428)

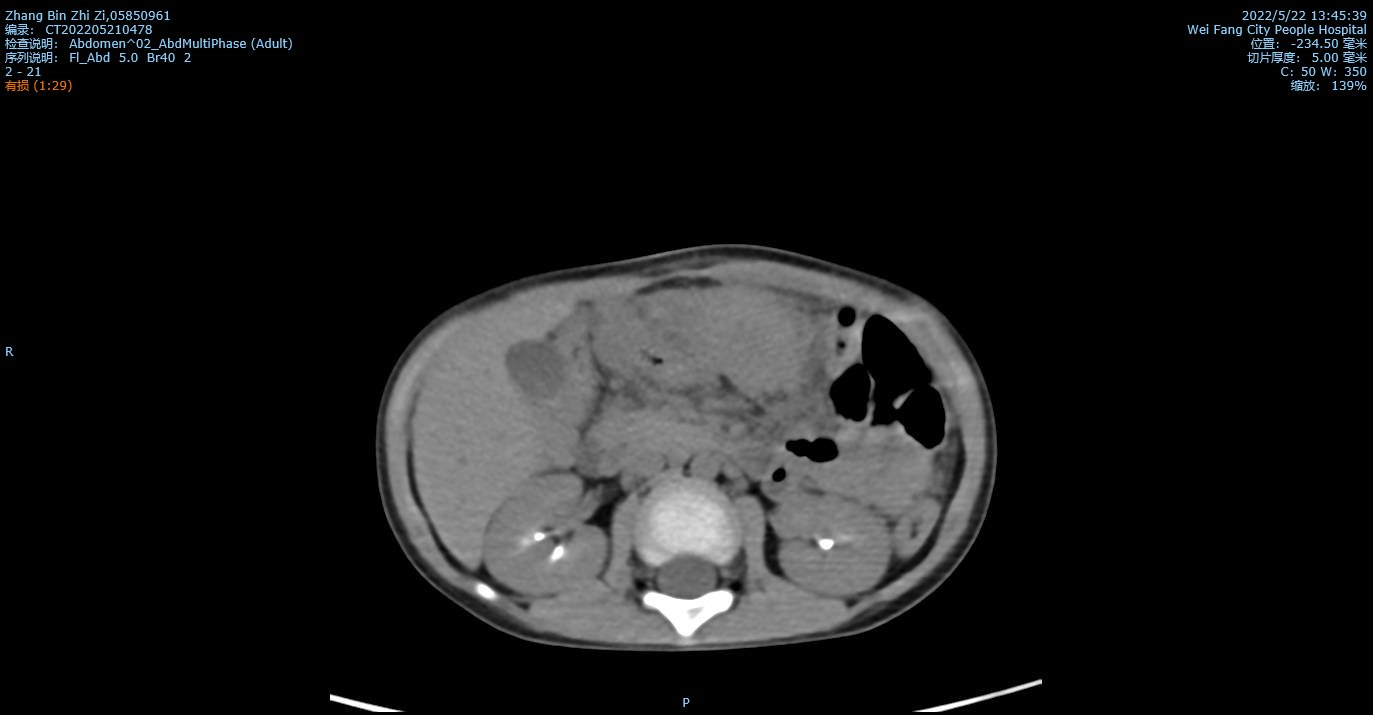

Supplement: Supplementary file 1 [file Datasheet1.zip › rawdata/1.png]

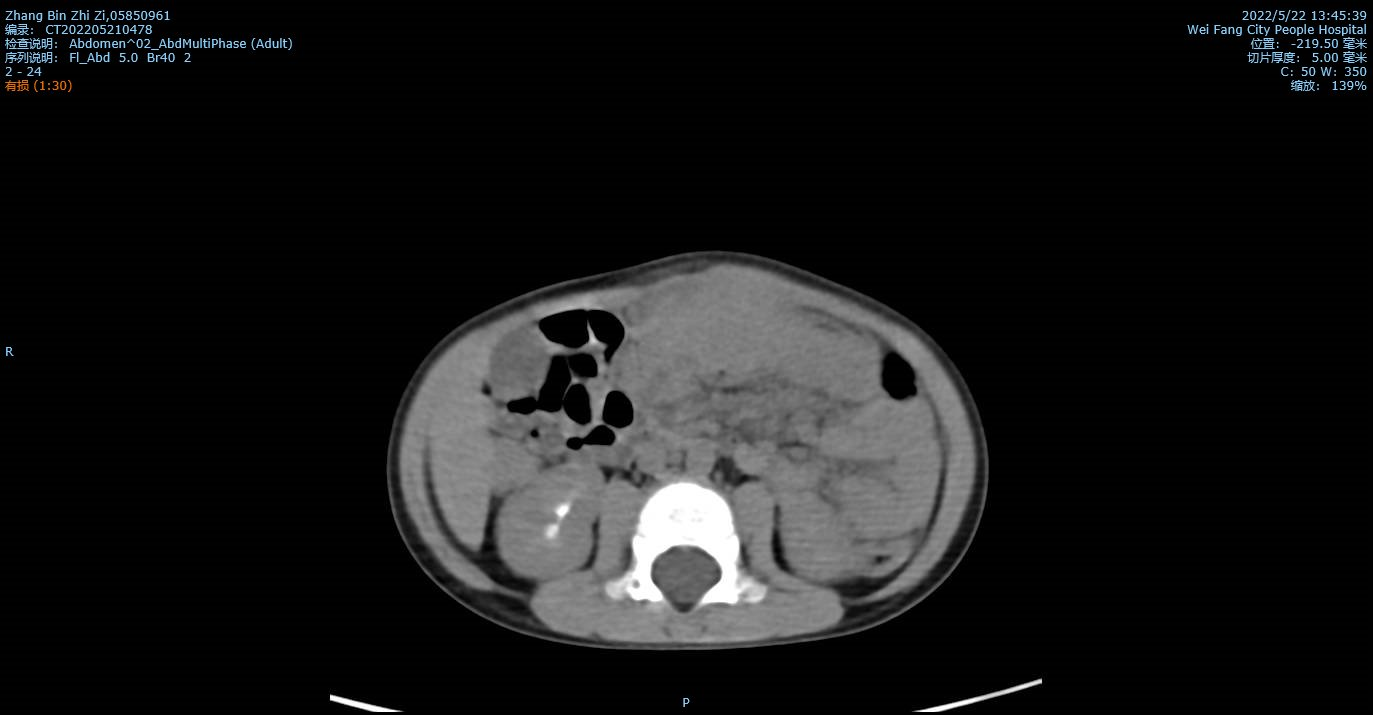

Supplement: Supplementary file 1 [file Datasheet1.zip › rawdata/2.png]

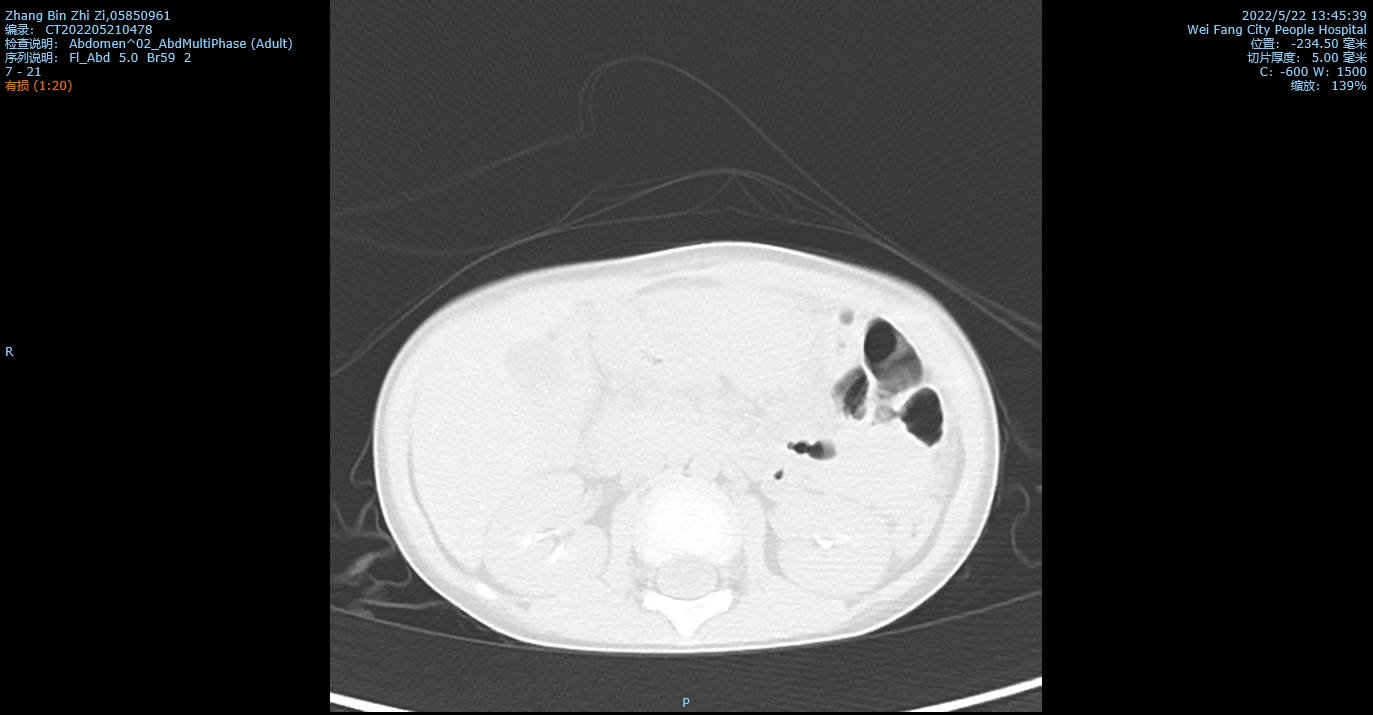

Supplement: Supplementary file 1 [file Datasheet1.zip › rawdata/3.png]

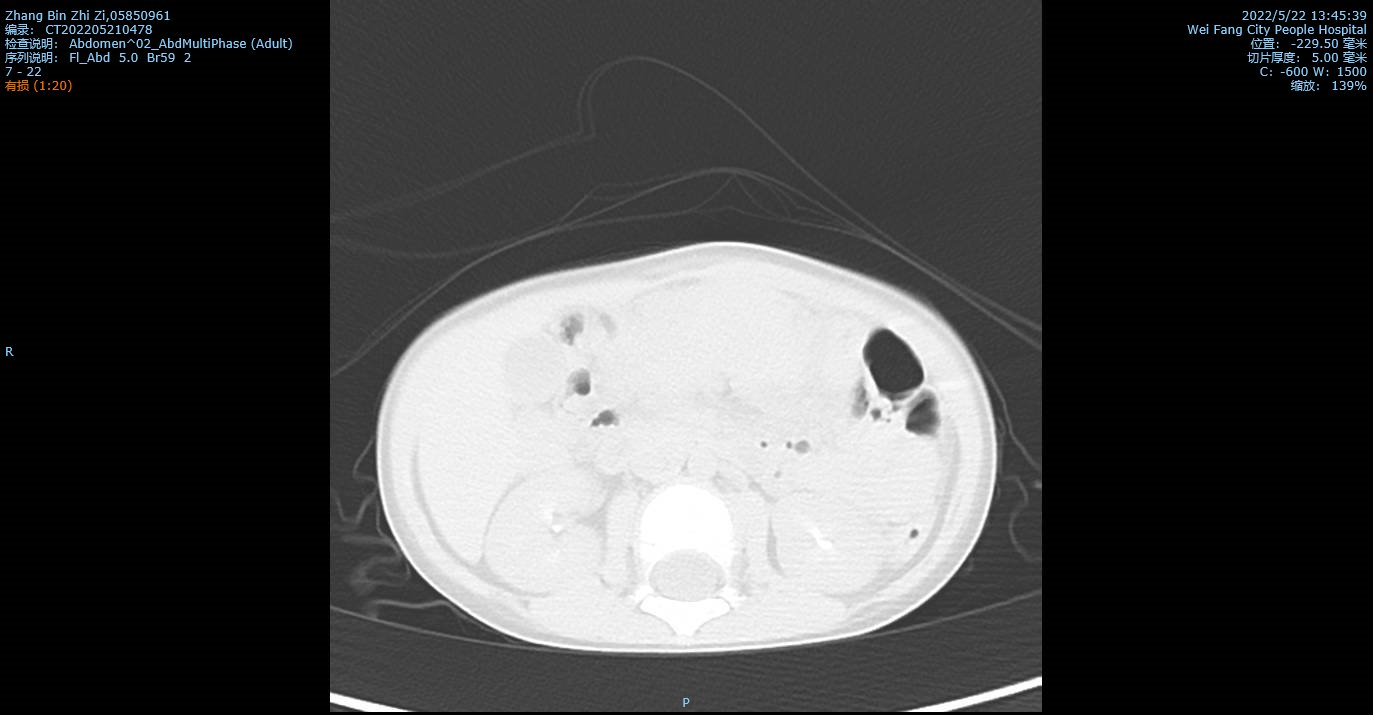

Supplement: Supplementary file 1 [file Datasheet1.zip › rawdata/4.png]

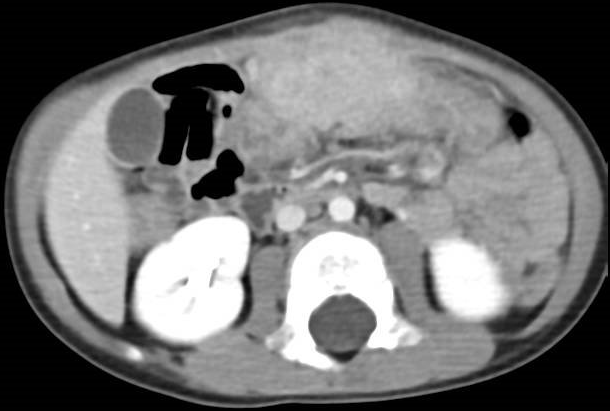

Supplement: Supplementary file 1 [file Datasheet1.zip › rawdata/5.png]

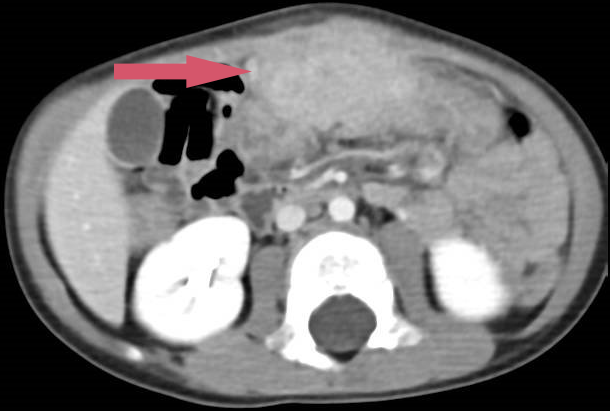

Supplement: Supplementary file 1 [file Datasheet1.zip › rawdata/5修改后.tif]

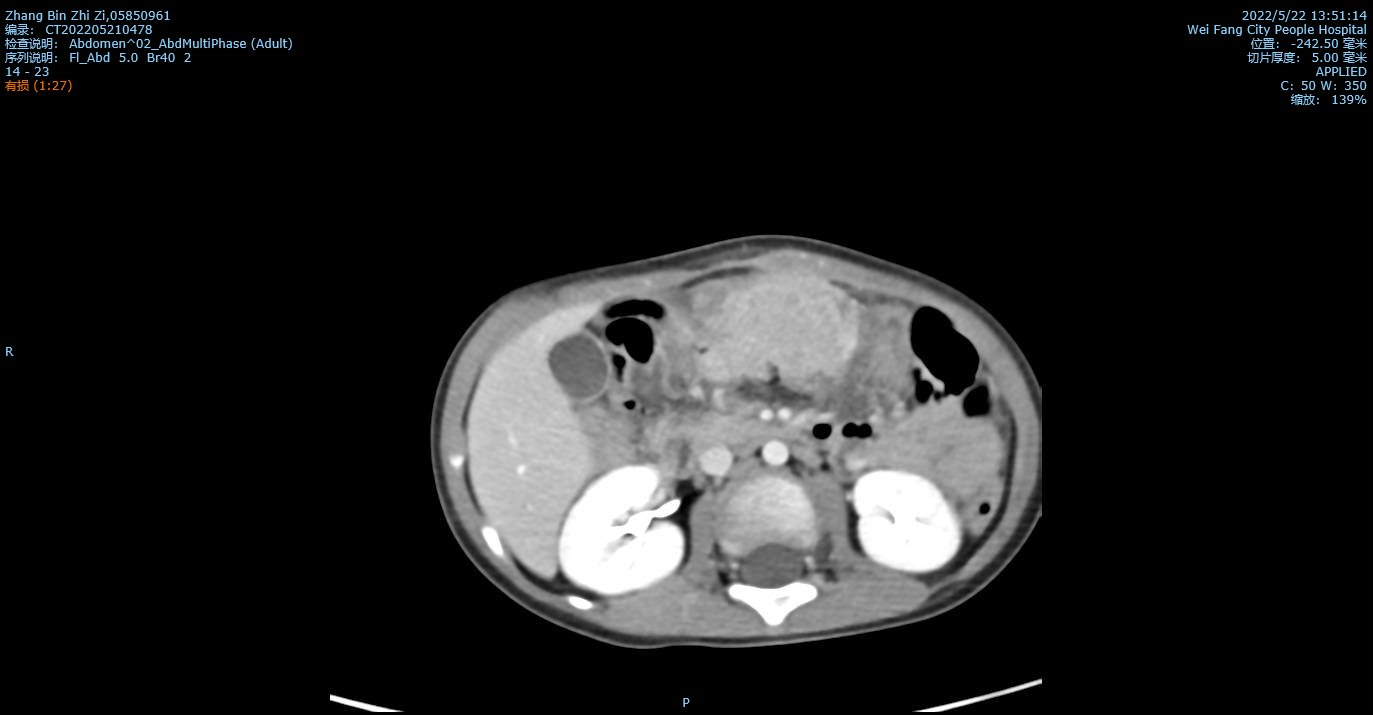

Supplement: Supplementary file 1 [file Datasheet1.zip › rawdata/6.png]

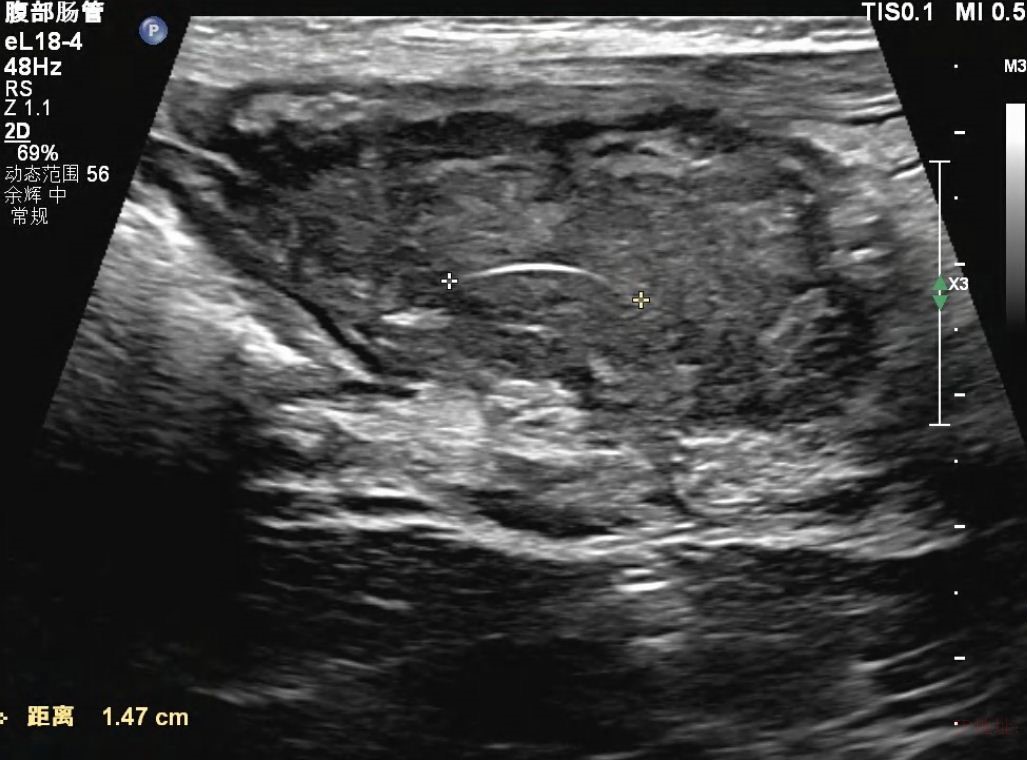

Supplement: Supplementary file 1 [file Datasheet1.zip › rawdata/张亦周 彩超1.JPG]

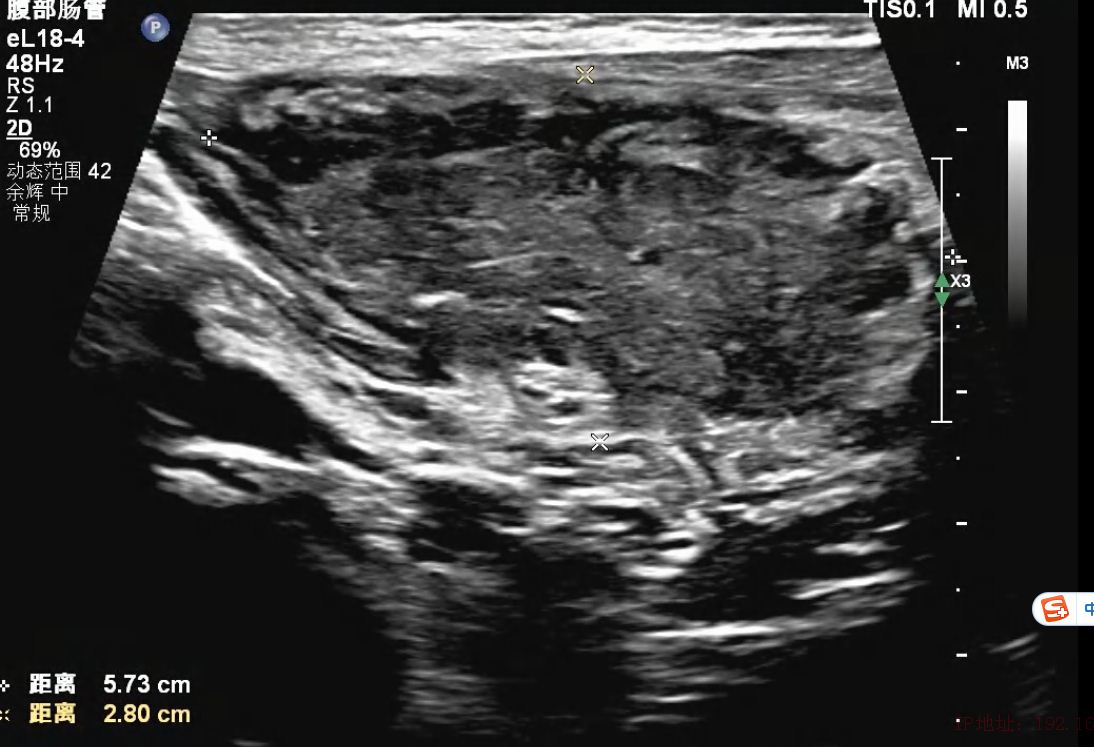

Supplement: Supplementary file 1 [file Datasheet1.zip › rawdata/张亦周 彩超2.JPG]

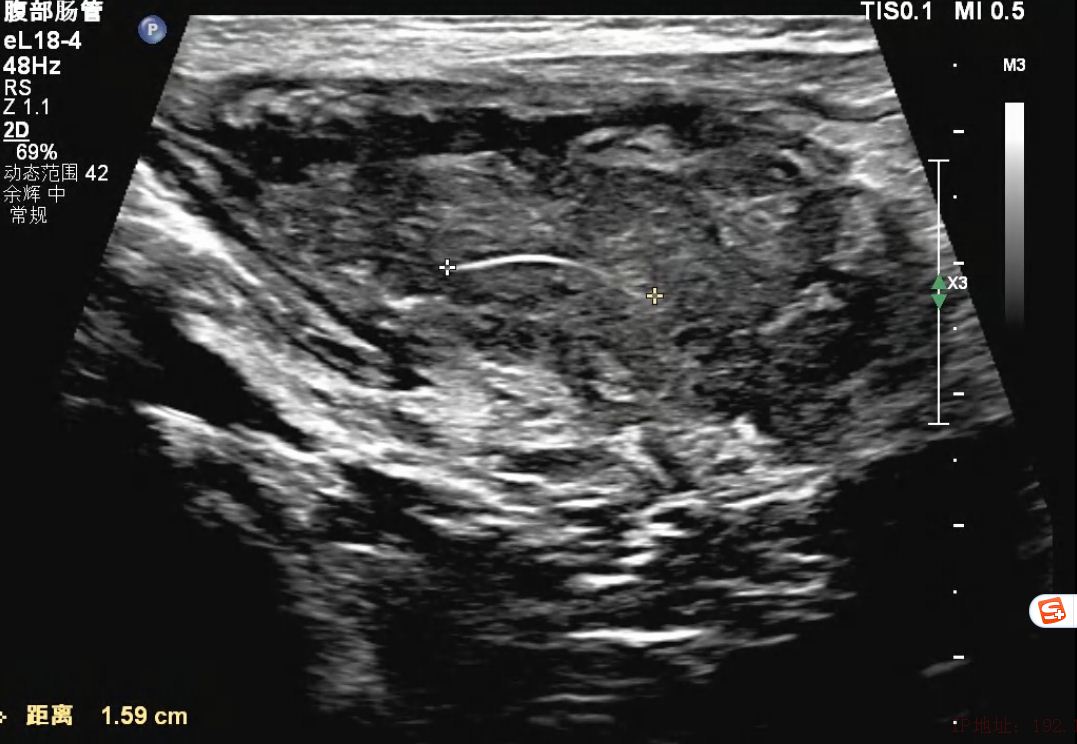

Supplement: Supplementary file 1 [file Datasheet1.zip › rawdata/张亦周 彩超3.JPG]

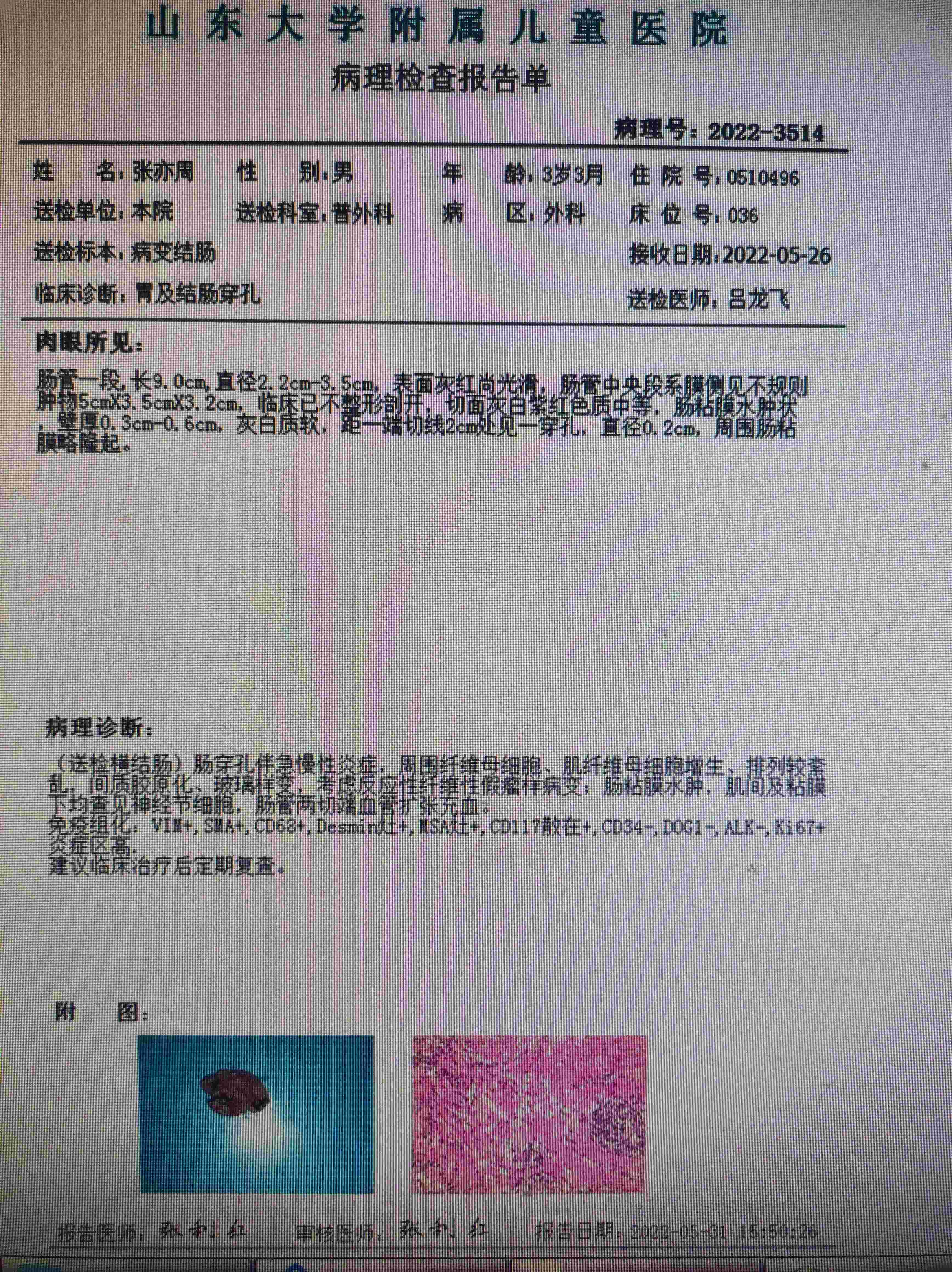

Supplement: Supplementary file 1 [file Datasheet1.zip › rawdata/微信图片_20240827155648.jpg]

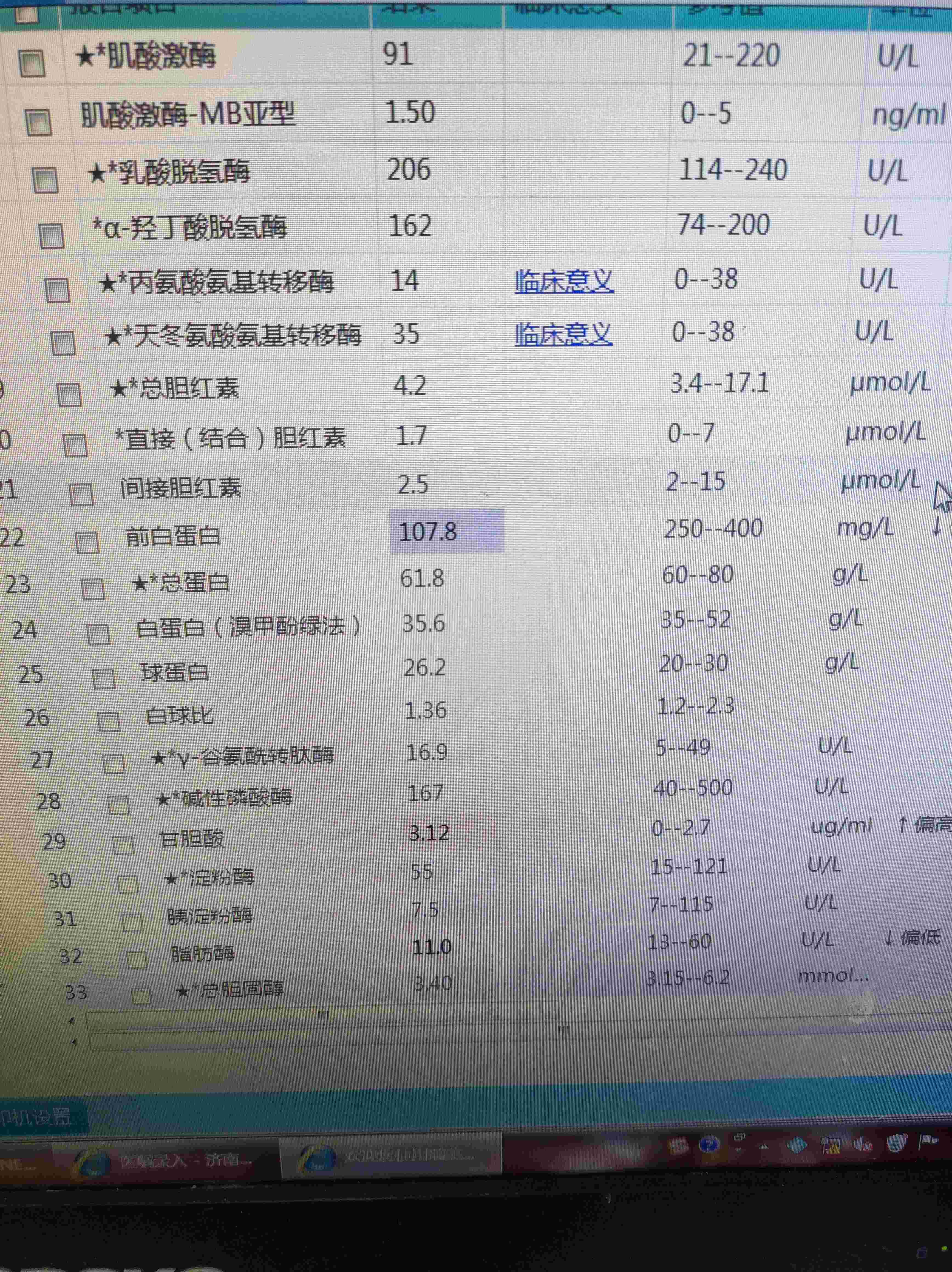

Supplement: Supplementary file 1 [file Datasheet1.zip › rawdata/微信图片_20240827155707.jpg]

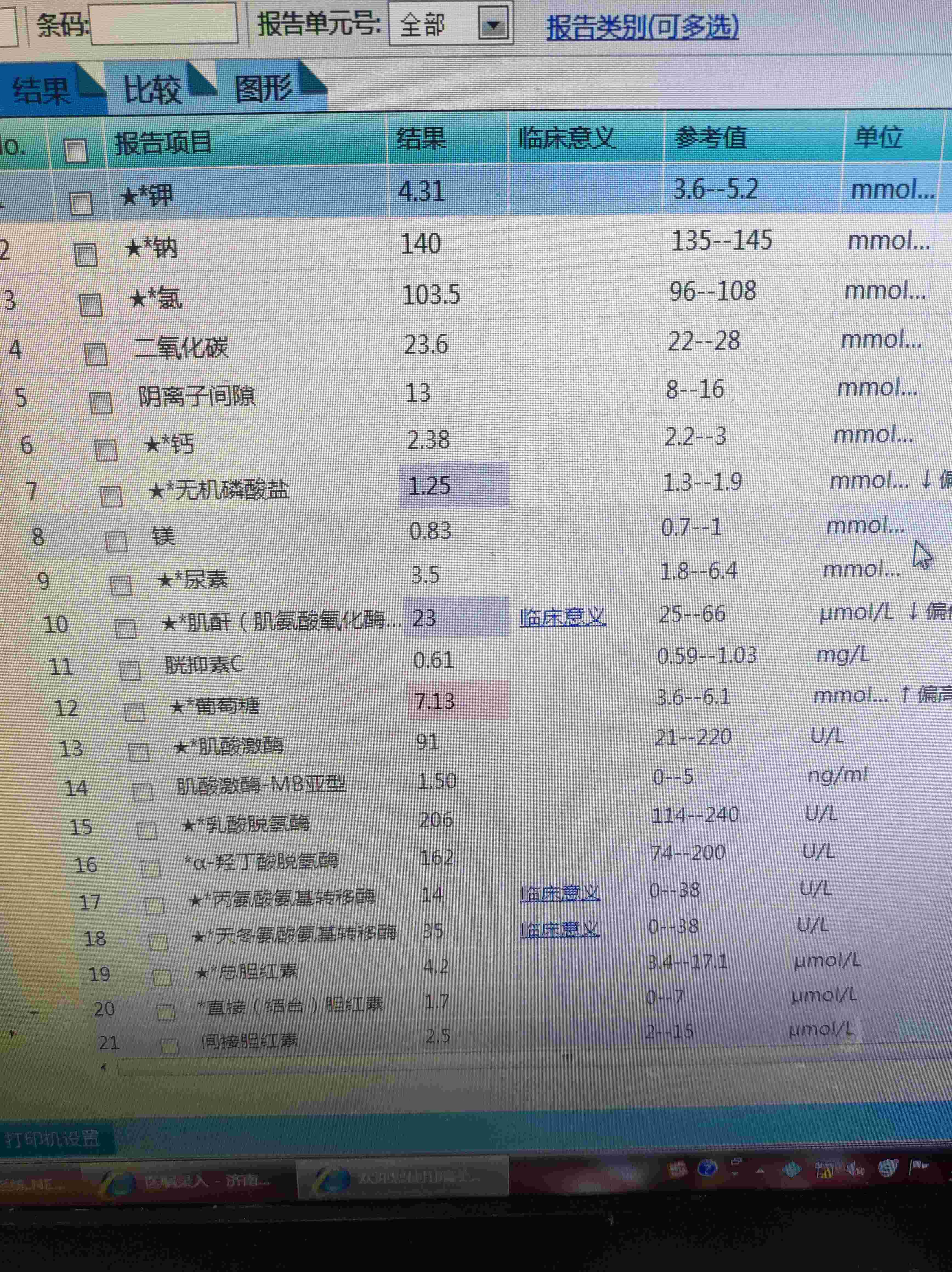

Supplement: Supplementary file 1 [file Datasheet1.zip › rawdata/微信图片_20240827155713.jpg]

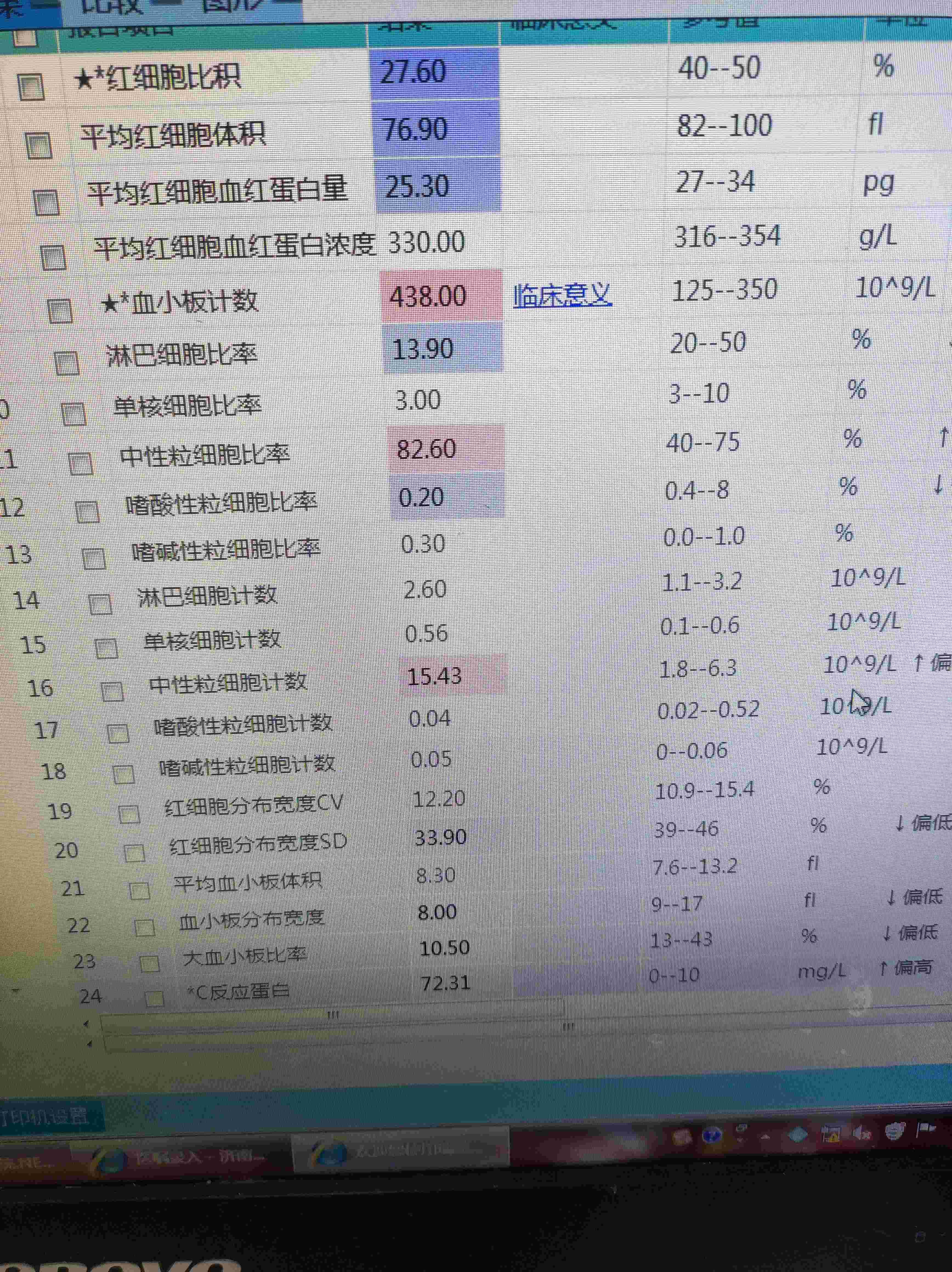

Supplement: Supplementary file 1 [file Datasheet1.zip › rawdata/微信图片_20240827155718.jpg]

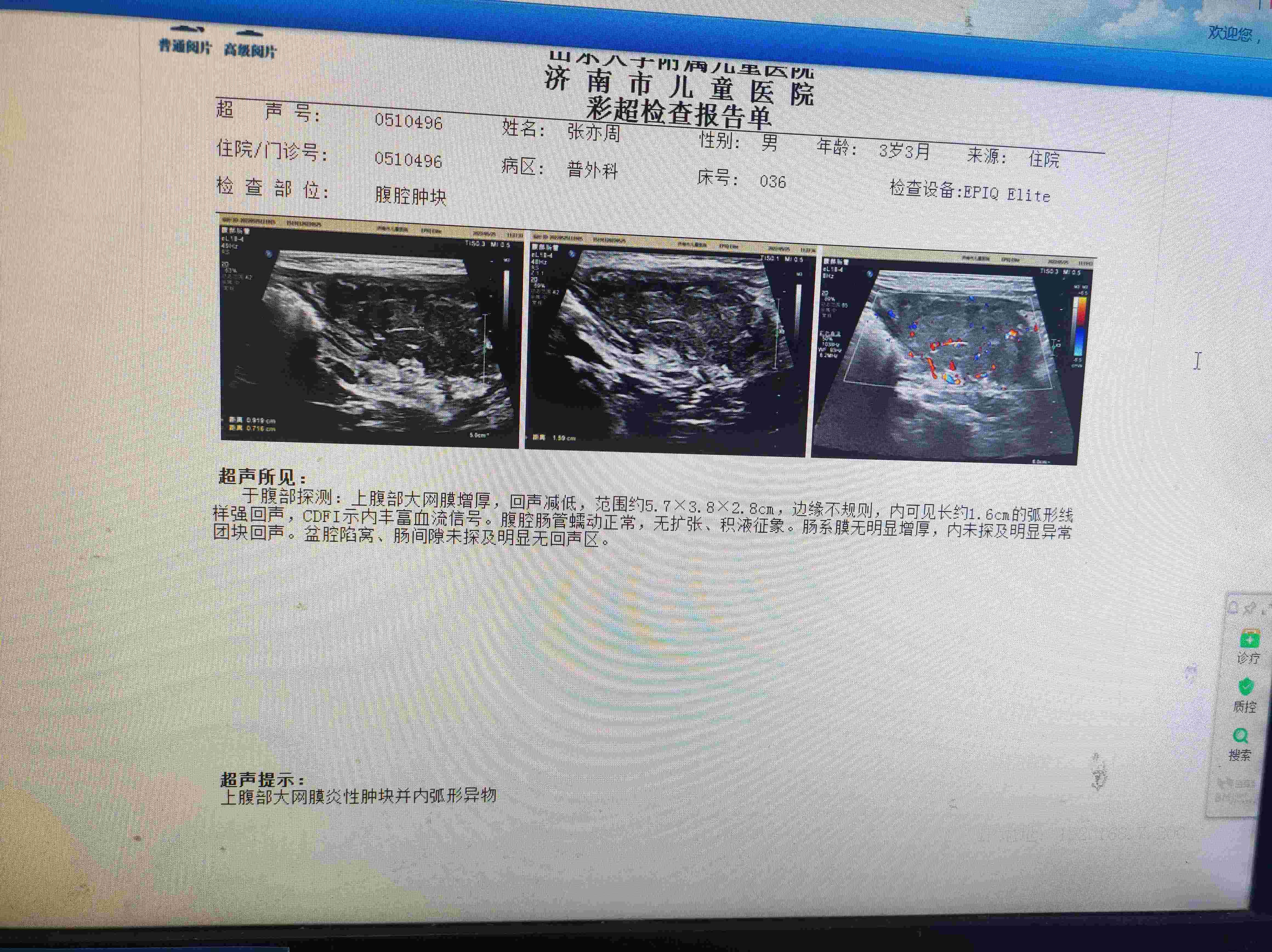

Supplement: Supplementary file 1 [file Datasheet1.zip › rawdata/微信图片_20240827155722.jpg]

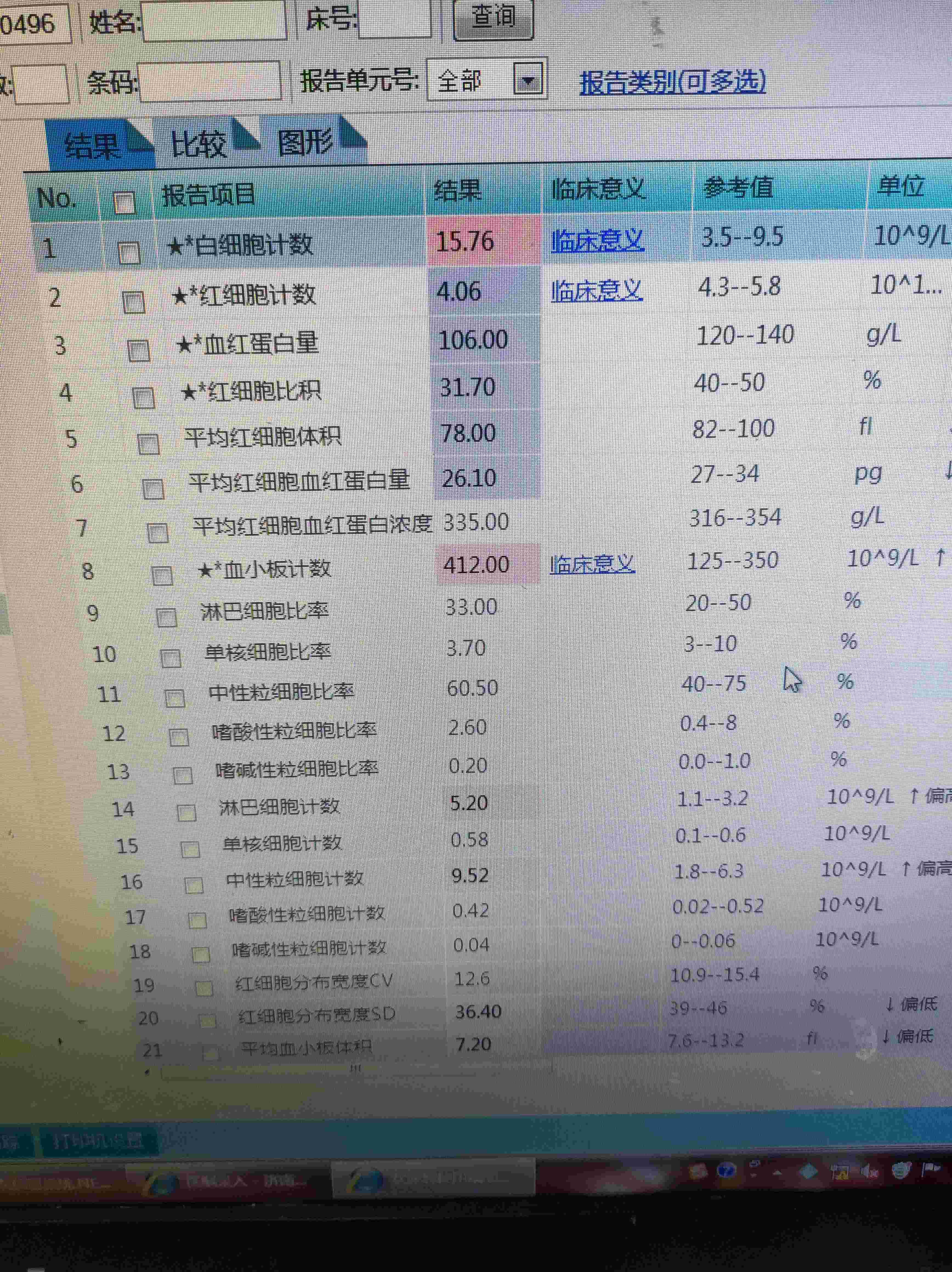

Supplement: Supplementary file 1 [file Datasheet1.zip › rawdata/微信图片_20240827155727.jpg]

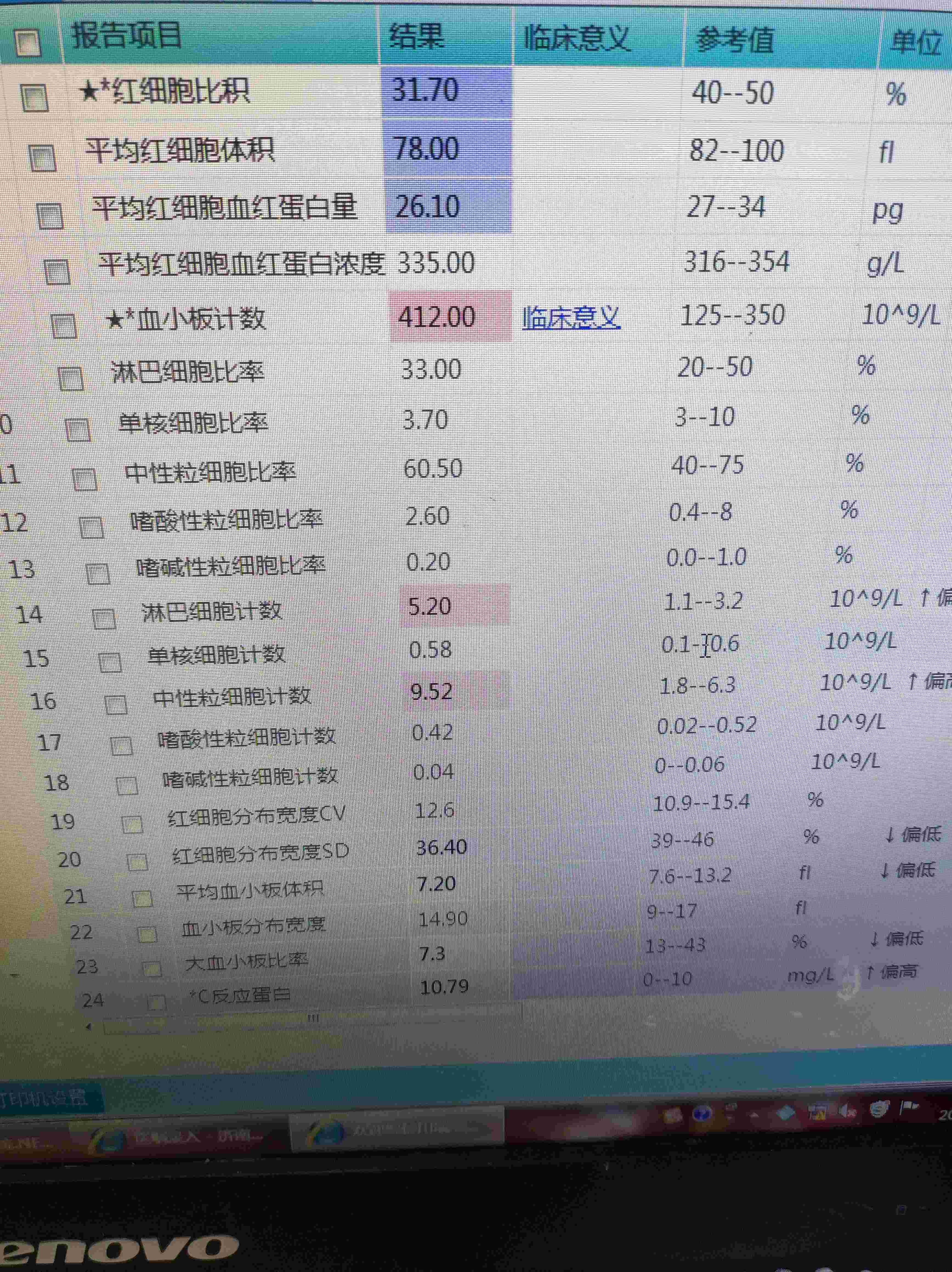

Supplement: Supplementary file 1 [file Datasheet1.zip › rawdata/微信图片_20240827155732.jpg]

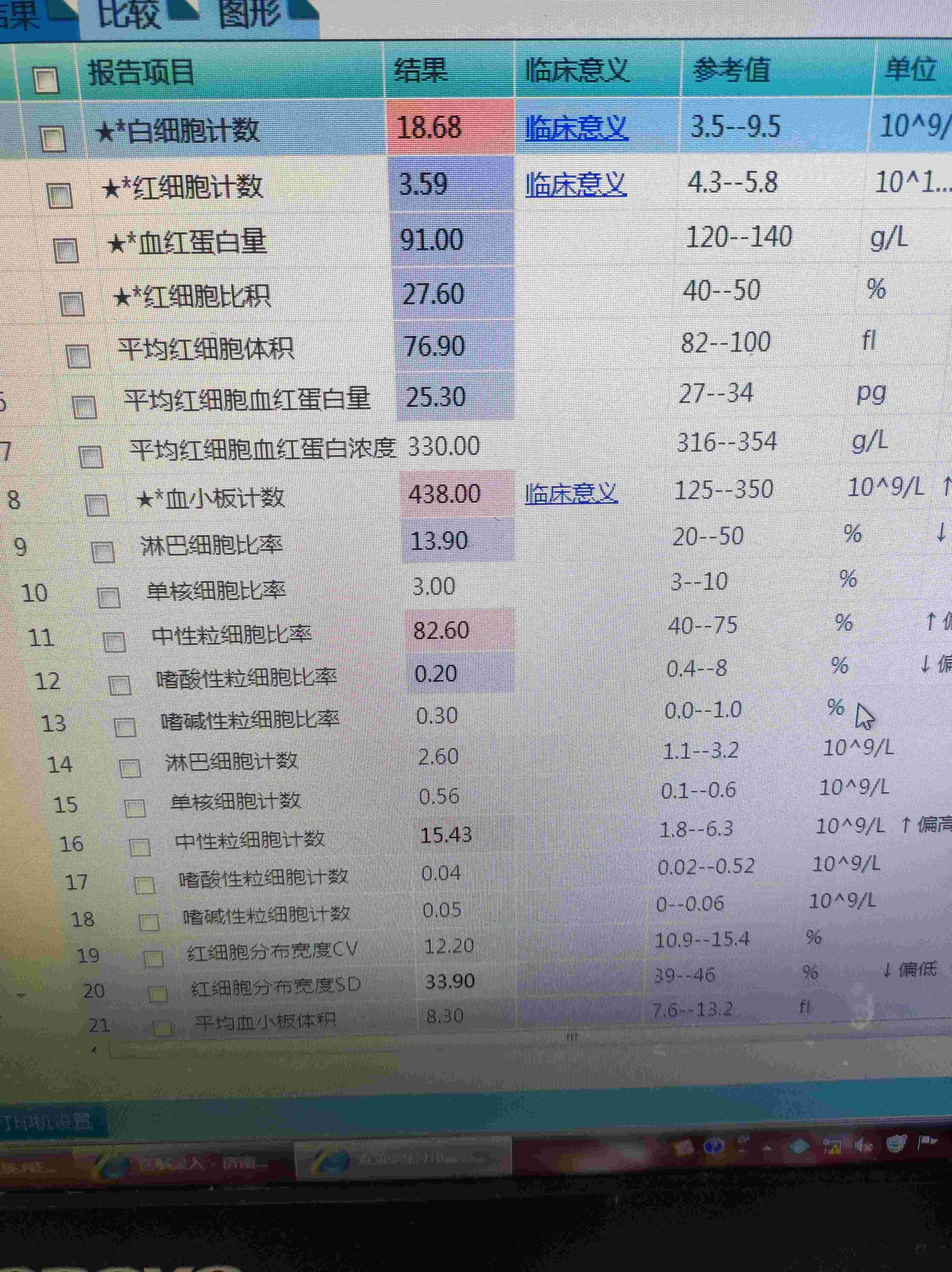

Supplement: Supplementary file 1 [file Datasheet1.zip › rawdata/微信图片_20240827155742.jpg]

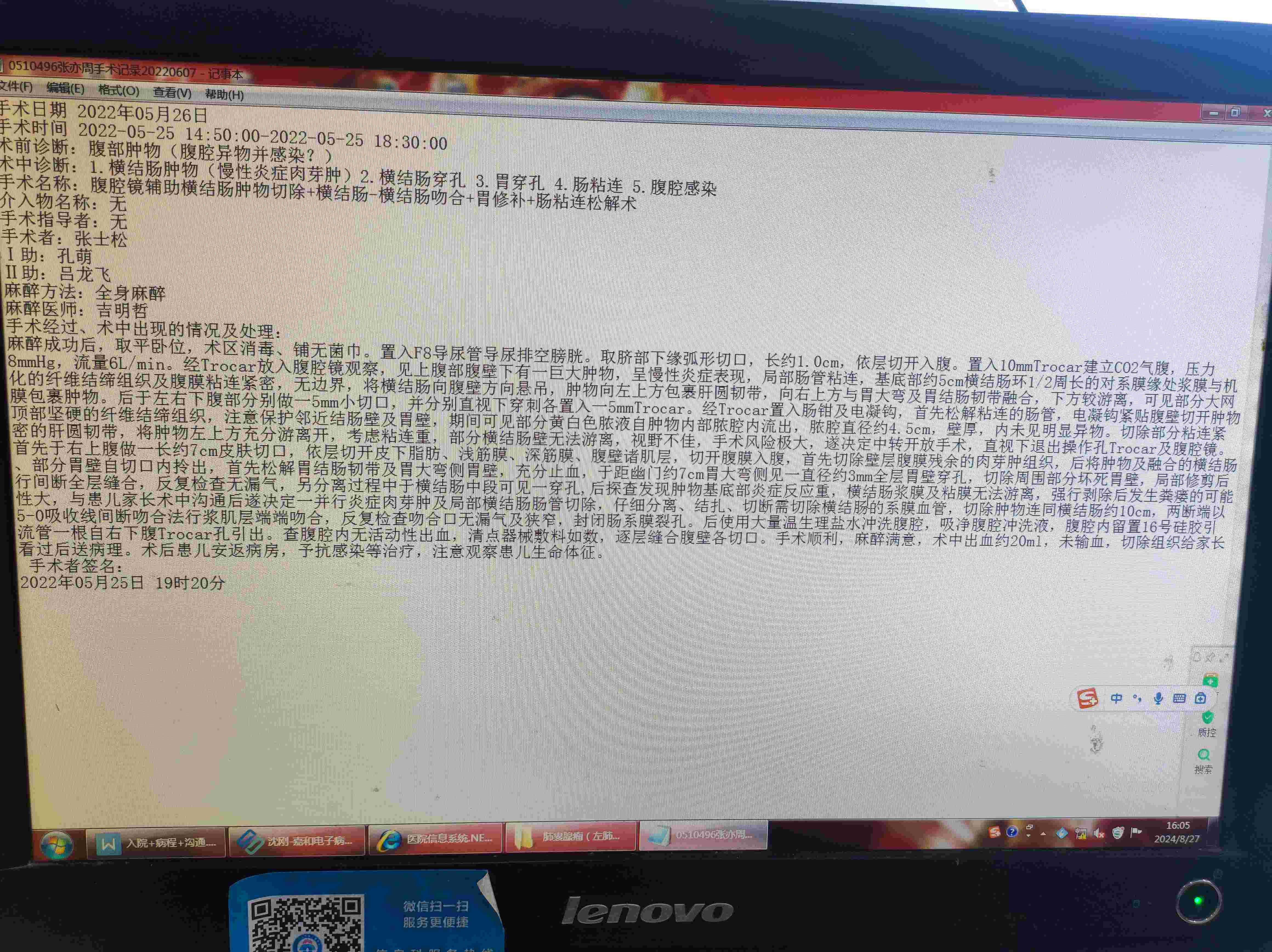

Supplement: Supplementary file 1 [file Datasheet1.zip › rawdata/微信图片_20240827162937.jpg]

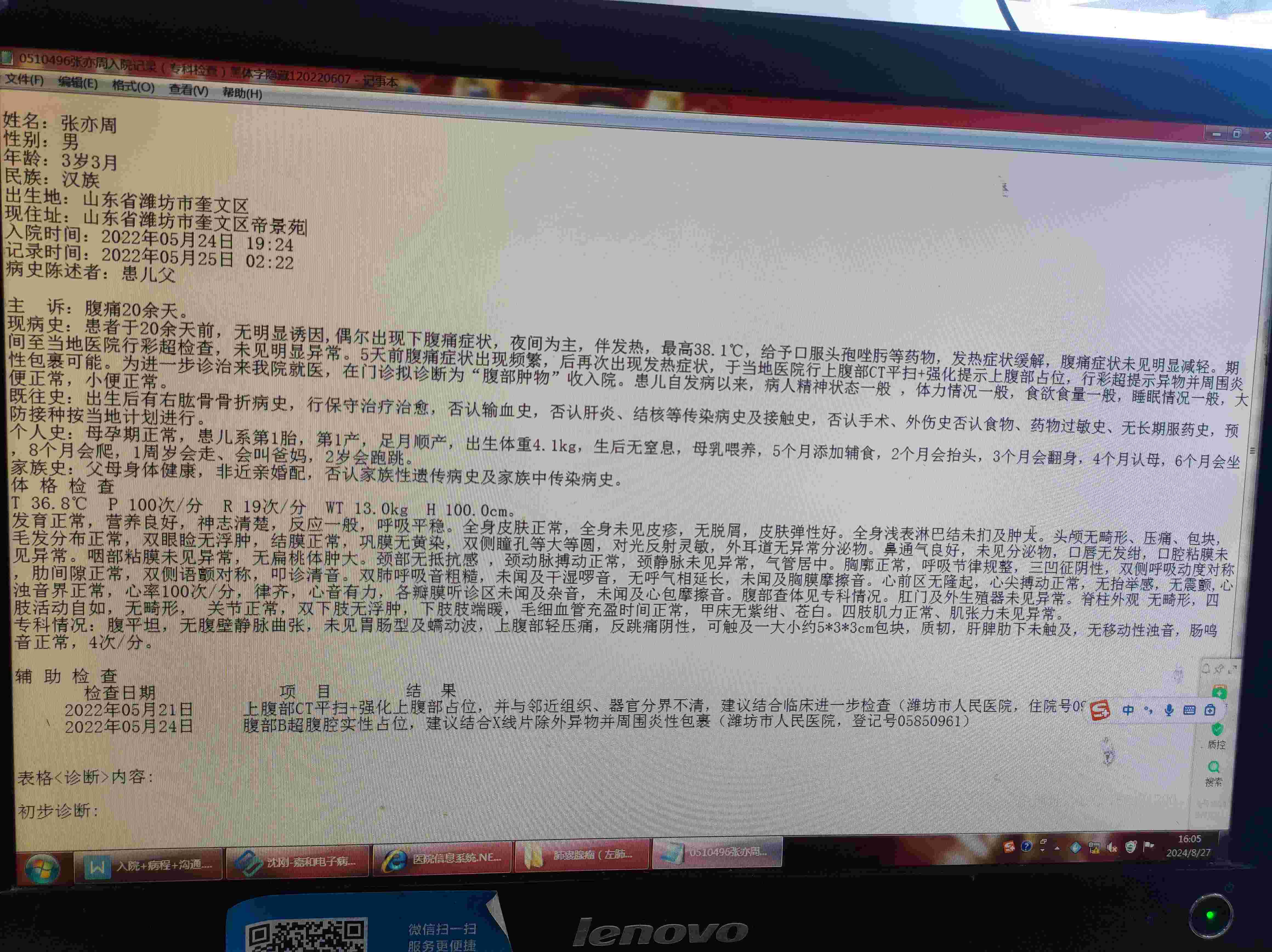

Supplement: Supplementary file 1 [file Datasheet1.zip › rawdata/微信图片_20240827162945.jpg]

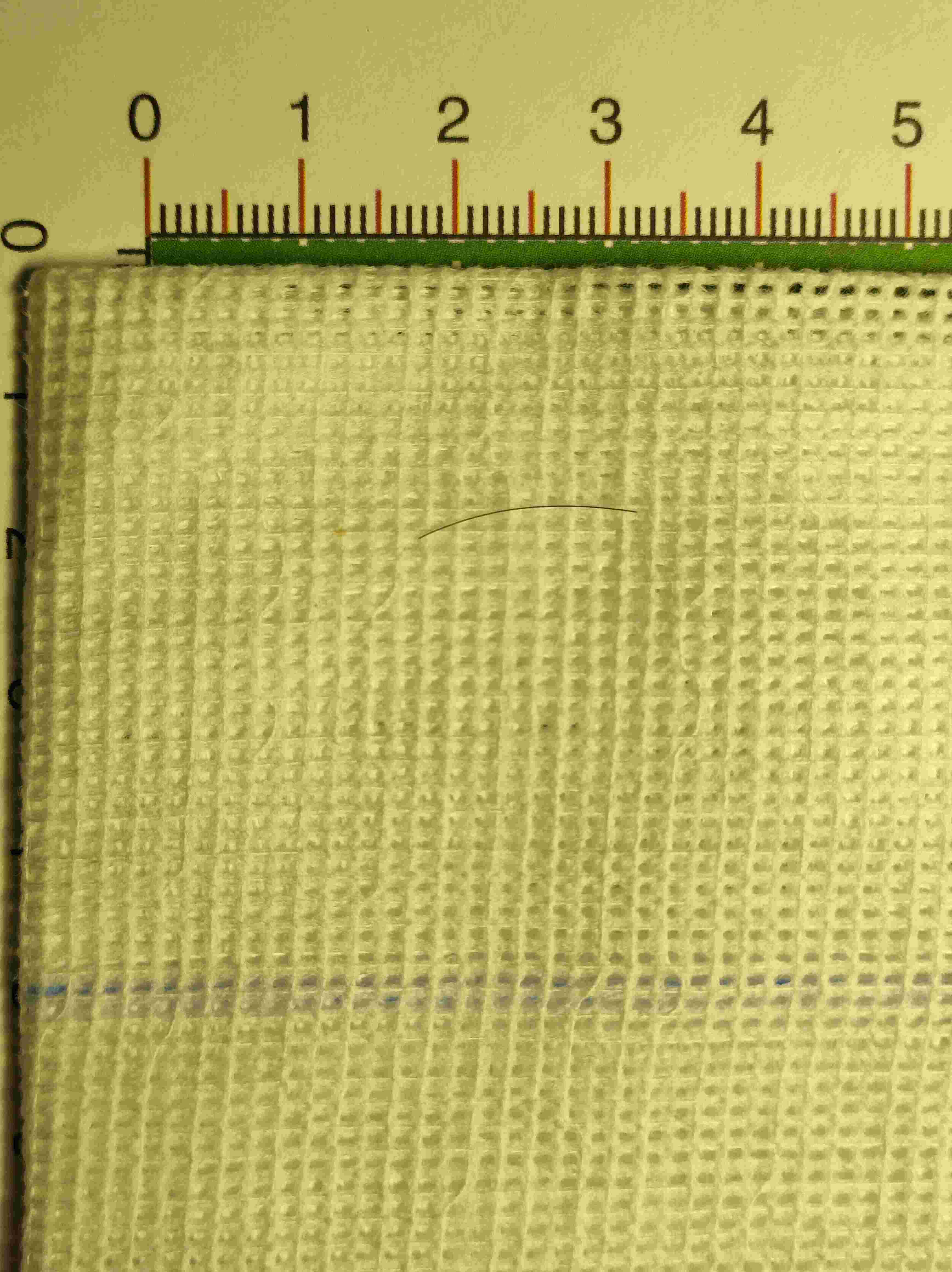

Supplement: Supplementary file 1 [file Datasheet1.zip › rawdata/微信图片_20241017153257.jpg]

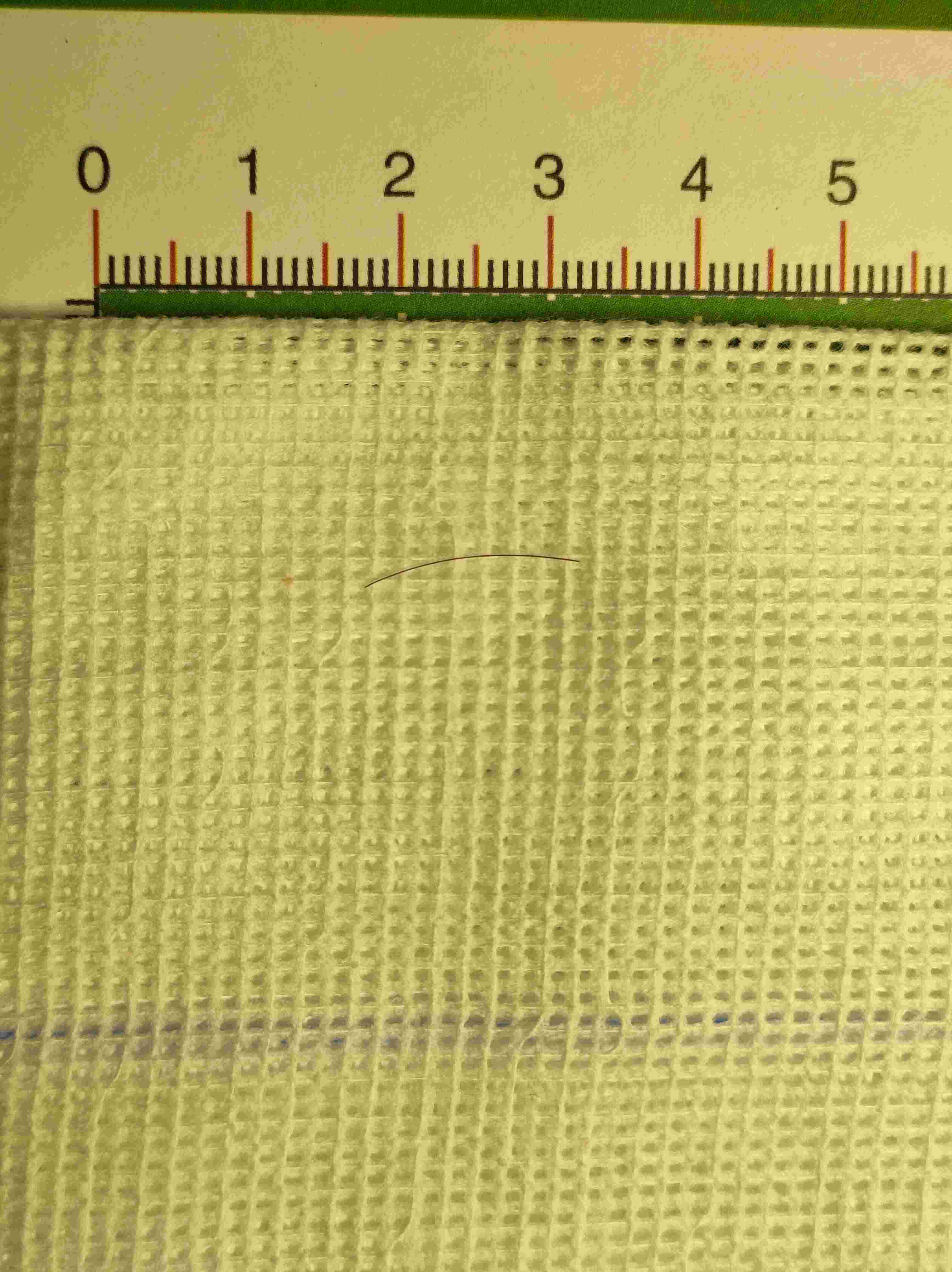

Supplement: Supplementary file 1 [file Datasheet1.zip › rawdata/微信图片_20241017153326.jpg]

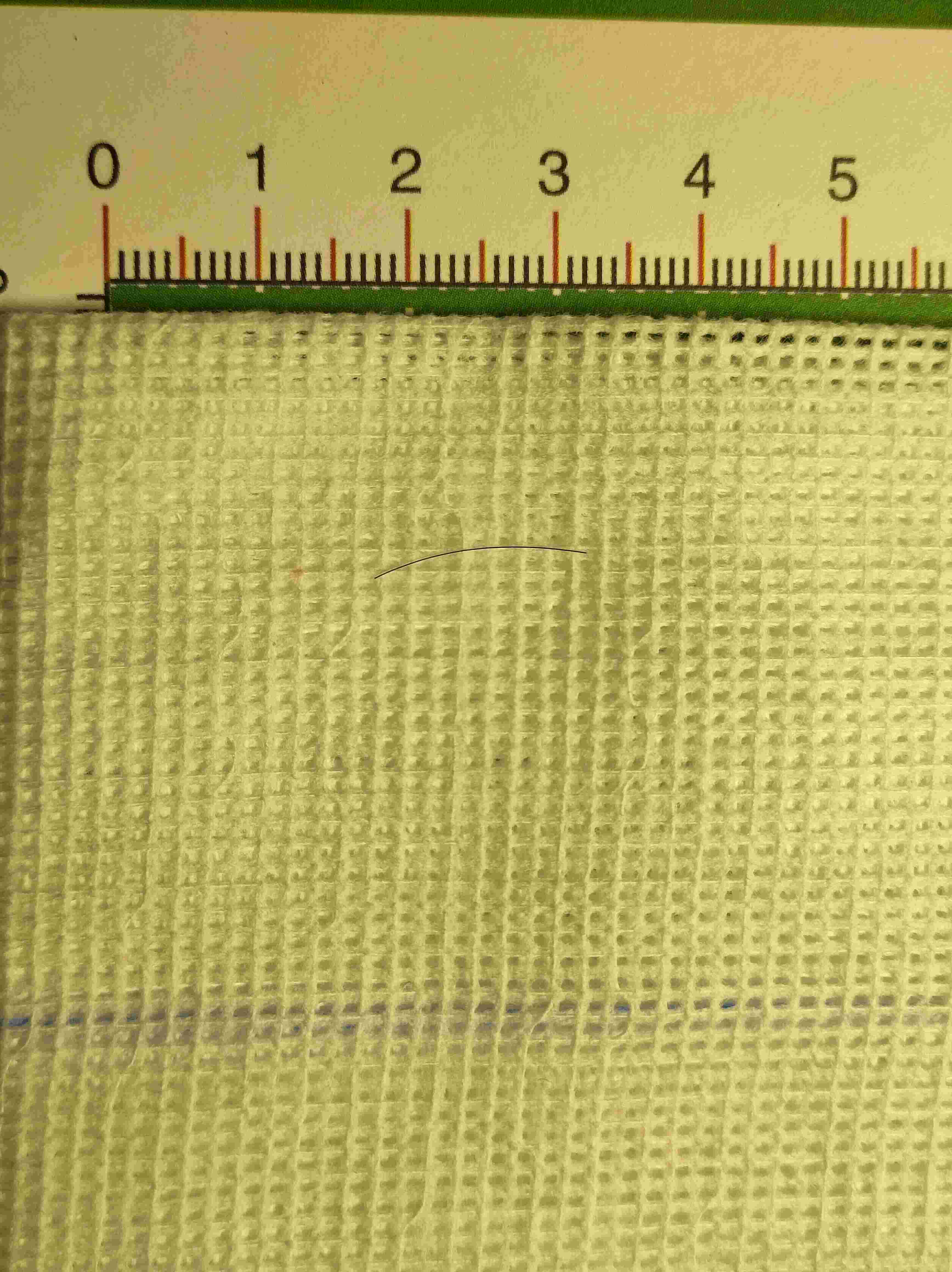

Supplement: Supplementary file 1 [file Datasheet1.zip › rawdata/微信图片_20241017153332.jpg]

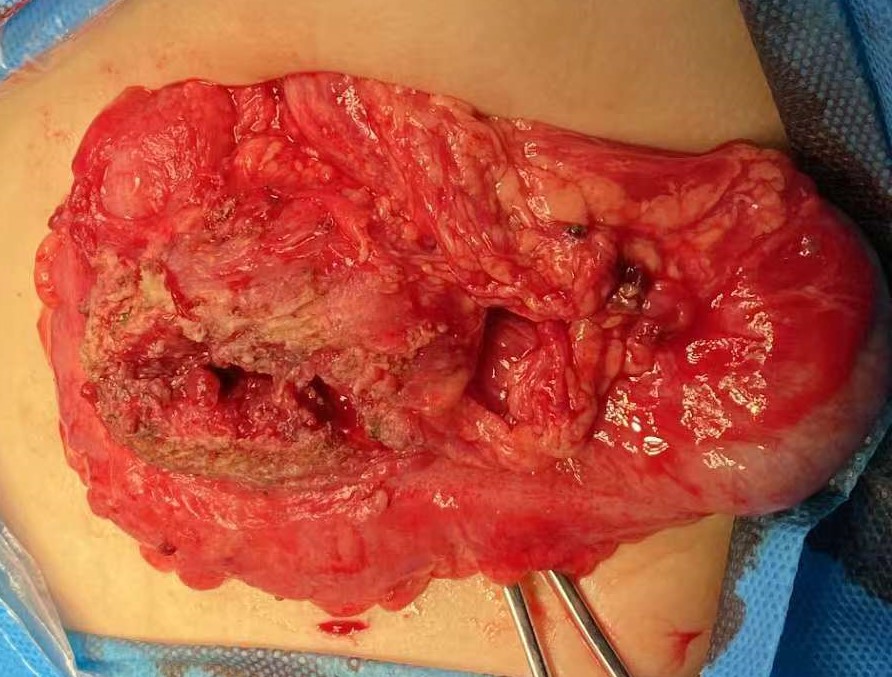

Supplement: Supplementary file 1 [file Datasheet1.zip › rawdata/术中图片1.jpg]

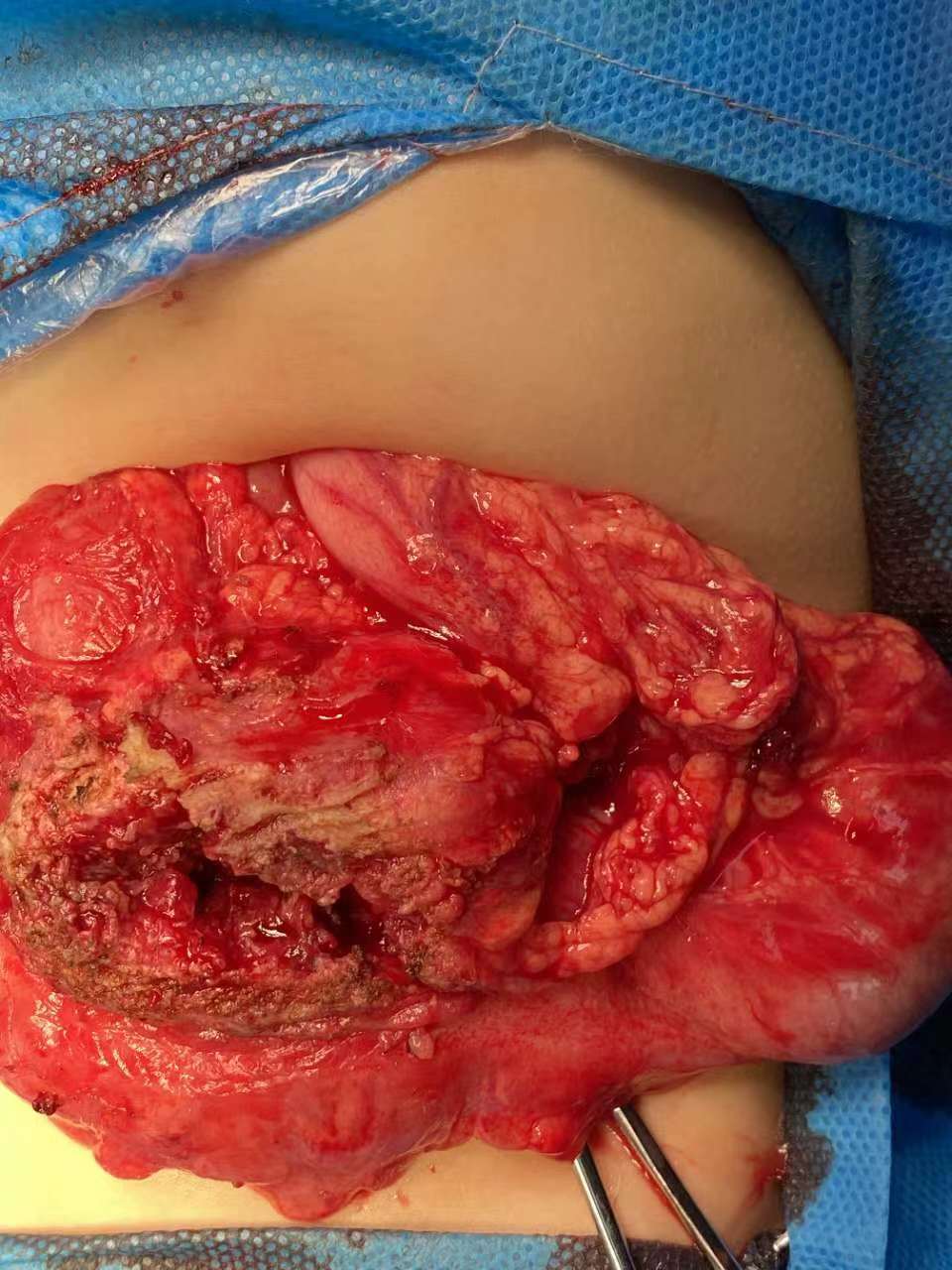

Supplement: Supplementary file 1 [file Datasheet1.zip › rawdata/术中图片2.jpg]
